# Introducing the PARCH Scale for Quantifying the Hydropathy of Nucleic Acids and Nucleic Acid–Protein Complexes

**DOI:** 10.1021/acs.langmuir.5c01051

**Published:** 2025-06-18

**Authors:** Ratnakshi Mandal, Jingjing Ji, Claire Nicole Sheridan, Anna M. Baur, Andre Christophe Noel, Shikha Nangia

**Affiliations:** Department of Biomedical and Chemical Engineering, 2029 Syracuse University, Syracuse, New York 13244, United States

## Abstract

Hydropathy studies have been extensively conducted for proteins, offering valuable insights into their structure and functionality. However, there is far less understanding of the hydropathy associated with the tertiary and quaternary structures of nucleic acids—such as deoxyribonucleic acid (DNA) and ribonucleic acid (RNA)—and their interactions with proteins. In this work, we extend our recently developed Protocol for Assigning a Residue’s Character on a Hydropathy (PARCH) scale to nucleic acids and nucleic acid–protein complexes. The PARCH scale quantifies the hydropathy of each nucleic acid residue based on its chemical identity and topographical features. The PARCH analysis for both DNA and RNA reveals that the backbone, consisting of phosphate and sugar atoms, is significantly more hydrophilic than the nucleotide bases; backbone PARCH values are an order of magnitude higher than those of the bases. In DNA, distortions from the organized double-helical structure, such as base flipping or altered base pairing, increase the hydropathy values. With its greater structural complexity, RNA exhibits a broader range of hydropathy values than DNA, reflecting its increased interaction with water. Thus, based on the PARCH values, RNA is more hydrophilic than DNA on average. PARCH analysis of DNA–protein and RNA–protein complexes reveals intricate binding patterns, including interactions between charged amino acid residues and the hydrophilic nucleic acid backbone, as well as hydrophobic patches on proteins engaging with the hydrophobic grooves of nucleic acid bases. These findings highlight the potential of PARCH analysis to provide valuable insights into the underlying principles of nucleic acid–protein interactions. The PARCH scale shows promise as a useful tool for advancing the development of functional RNA and DNA fragments for future therapeutic applications.

## Graphical abstract

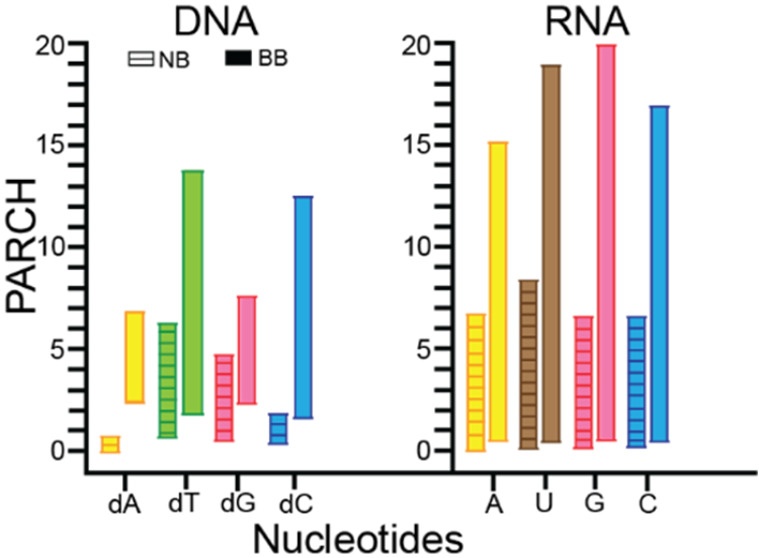

## Introduction

Understanding the hydropathy of nucleic acids—deoxyribonucleic acid (DNA) and ribonucleic acid (RNA)—is essential for unraveling their critical roles and behaviors in living systems. The term hydropathy was introduced by Kyte and Doolittle in the context of a numerical scale^1^ that measures the relative hydrophobicity or hydrophilicity of amino acid residues based on experimental observations. Each amino acid is assigned a value reflecting its tendency to be found in hydrophobic (water-avoiding) or hydrophilic (water-attracting) environments. Despite certain limitations, the Kyte–Doolittle scale remains widely used to predict membrane-spanning regions, surface accessibility, and protein folding patterns by analyzing the hydropathy profiles along protein sequences. However, this scale is not applicable to nucleic acids, underscoring the need for a distinct and tailored approach to quantifying hydropathy in DNA and RNA.

Quantifying the hydropathy of nucleic acids presents unique challenges due to their chemically distinct components (nitrogenous bases, pentose sugars, and charged phosphate groups) connected via phosphodiester bonds into strands that hierarchically organize into primary, secondary, tertiary, and quaternary structures. While extensive hydropathy scales have been developed for proteins over the past five decades,^1–13^ the same level of attention has not been given to nucleic acids.^14^ Currently, there is no unified scale capable of quantifying the relative hydrophilicity or hydrophobicity of both nucleotides and amino acids. This study addresses this gap by extending the Protocol for Assigning a Residue’s Character on a Hydropathy (PARCH)^15^ scale, initially developed for proteins, to nucleic acids.

The hydropathy of nucleic acids is intricately tied to their structural hierarchy, with each level contributing distinct characteristics that influence biological function. The primary structure of nucleic acids is a linear sequence of nucleotides;^16^ however, chemical differences between DNA and RNA—specifically, the presence of deoxyribose in DNA and ribose in RNA—lead to distinct hydropathy profiles in their backbones. The secondary structure involves localized folding patterns, such as the canonical double helix of DNA, stabilized by hydrogen bonds between complementary base pairs.^17^ In contrast, the RNA secondary structures,^18^ include hairpins, loops, bulges, and stems, essential for diverse cellular processes like splicing, translation, and ribozyme catalysis.

Tertiary structures form through folding interactions between secondary elements, producing complex three-dimensional configurations that are critical for function. DNA tertiary structures enable supercoiling, which is essential for efficient packaging within the nucleus,^19,20^ while RNA tertiary structures facilitate specific functional roles, such as rRNA folding in protein synthesis.^21^ At the quaternary level, nucleic acids interact with proteins or other nucleic acids to form higher-order assemblies, such as nucleosomes in chromatin^22^ or RNA–protein complexes like the ribosome^23^ and spliceosome.^24^ These structures regulate gene expression,^25^ transcription,^26^ and cellular organization,^22^ highlighting the need for a comprehensive understanding of their hydropathy.

The structural flexibility of nucleic acids significantly contributes to the complexity of their hydropathy. DNA can transition among A-form, B-form, and Z-form helices, with these conformational changes influenced by factors such as nucleotide sequence, pH, ionic composition, solvent conditions, and temperature.^27^ Similarly, RNA predominantly adopts a right-handed A-form helix but can transition to alternative conformations in specific sequence contexts.^28^ These structural variations profoundly affect the hydropathy of nucleic acids, shaping their interactions with proteins, small molecules, and other nucleic acids.

Moreover, the higher-order interactions are critical for cellular functions, highlighting the importance of understanding quaternary structures to elucidate the mechanisms of gene regulation and cellular organization. In DNA, interactions with proteins are crucial for chromatin organization and gene regulation. For instance, nucleosomes, where DNA is wrapped around histone proteins, form the fundamental units of chromatin. Chromatin further organizes into higher-order structures,^22^ including loops and domains, influencing gene accessibility^25^ and transcriptional activity.^26^ For RNA, quaternary structures involve its assembly with proteins into functional complexes such as the spliceosome, which is responsible for RNA splicing,^29^ and the ribosome, which is essential for protein synthesis.^21^ By quantifying hydropathy at each structural level, our study underscores the central role of nucleic acids in storing, transmitting, and regulating genetic information within cells.

Building on our previous development of the PARCH scale for proteins, where we analyzed over 270,000 amino acids from more than 1000 proteins,^30^ we aim to apply a similar approach to nucleic acids. The PARCH scale accounts for the local geometry and nanoscale topography surrounding residues, providing context-dependent hydropathy values that enhance accuracy. Our findings demonstrated that amino acids have no fixed hydropathy values, as their local chemical environments significantly influence their hydropathy.

Despite the importance of nucleic acid–protein interactions in cellular processes, no current method integrates chemistry and topography to analyze nucleic acids’ hydropathy or compares nucleic acids and proteins on the same scale. By quantifying the hydropathy of nucleic acids using an extended PARCH scale, we aim to bridge this gap. Our approach offers new insights into the complex interactions between nucleic acids and proteins, moving beyond simplistic electrostatic categorizations. These interactions underpin critical cellular processes, making their detailed study essential for advancing our understanding of molecular biology. Furthermore, our analyses have practical applications in nucleic acid recognition,^31^ self-assembly,^32,33^ liquid–liquid phase separation,^34–36^ and interfacial adsorption.^37,38^ By employing the PARCH scale, we can identify hydrophobic and hydrophilic regions in these synthetic structures, optimizing their design and functionality for various applications.

## Methods

The protocols and workflow to calculate the PARCH values for nucleotides are similar to amino acids in proteins, except that a nucleotide, being much larger than an amino acid, has two PARCH values: one for backbone phosphate and sugar atoms (BB) and the other for the bases (NB). The NB and BB components of the nucleotides are also chemically distinct. The BB with the phosphorylated sugar is charged and forms the structural framework of the strand, while NB forms specific pairs through hydrogen bonding with the complementary DNA strand to form the double-helical structure. The four DNA nucleotides (Figure 1), deoxyadenine (dA), deoxythymine (dT), deoxycytosine (dC), and deoxyguanine (dG), have PARCH values for the BB and NB components, written in (BB, NB) format. Similarly, the PARCH values of the four RNA nucleotides (Figure 1), adenine (A), uracil (U), guanine (G), and cytosine (C), are written in the (BB, NB) format.

**Figure 1:**
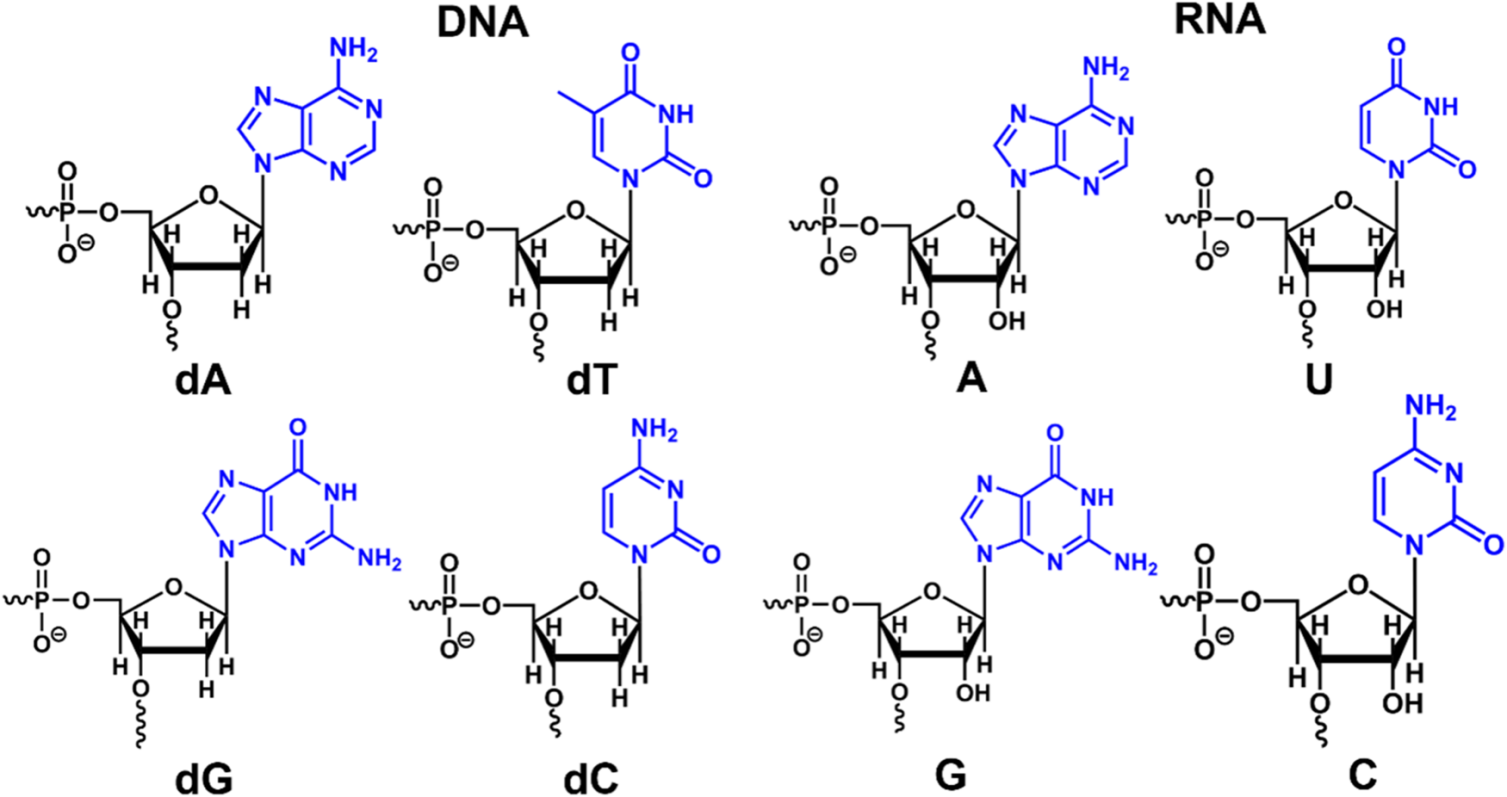
Chemical structures of DNA and RNA nucleotides. Each of the four DNA deoxyribonucleotides (dA, dC, dG, and dT) consists of a deoxyribose sugar and a phosphate group (BB, black), along with one of the four nitrogenous bases (NB). Similarly, the four RNA nucleotides (A, C, G, and U) include a ribose sugar (featuring a hydroxyl (−OH) group at the 2′ carbon instead of hydrogen (−H) as in deoxyribose), a phosphate group (BB, black), and one of the four nitrogenous bases (NB). The PARCH values of the BB and NB regions in all DNA and RNA nucleotides are labeled using the (BB, NB) format.

The PARCH value calculation for a nucleotide (*i*) involves computing the water count (*w*_*i*α_) around α = BB, NB regions of the residue at a given time (*t*). We map the water count to η_*i*α_(*t*) and compute the autocorrelation function *C*_*i*α_(τ), which is defined as

1 $$C_{i\alpha }\left(\tau \right)=\left\langle \eta_{i\alpha }\left(t\right)\eta_{i\alpha }\left(t+\tau \right)\right\rangle$$

Next, we compute the average of the autocorrelation function from the time integral

2 $${\mathrm{C̅}}_{i\alpha }=\frac{1}{\tau_{\max}}\int_{0}^{\tau_{\max}}d\tau C_{i\alpha }\left(\tau \right)$$

Finally, we compute the PARCH value using

3 $$PV_{i\alpha }=\frac{{\mathrm{C̅}}_{i\alpha }}{{\mathrm{C̅}}_{\mathrm{ref}}}\times 10$$

where *C̅*_ref_ is the reference value for a zwitterionic lysine amino acid. The multiplication factor of 10 is a scaling factor in case *C̅*_*i*α_/*C̅*_ref_ is a very small number. The parameters used for the PARCH value calculations in eqs 1–3 are provided in the following sections. Notably, we used zwitterionic lysine as a reference, enabling direct comparison of nucleic acids and proteins on a unified PARCH scale. Like in the proteins, if the BB or NB regions of the residue are embedded in the fold with no access to water, then *C̅*_*i*α_ *=* 0, and the PARCH values of the regions are zero.

### Simulated Systems

We performed PARCH analysis on experimentally determined DNA and RNA tertiary and quaternary structures.

1. **DNA fragments**. A set of 30 single- and double-stranded DNA structures, comprising 139 dA, 132 dC, 180 dG, and 146 dT (Table S1), were studied. These structures include duplexes composed of 14–24 residues with two complementary strands held together by hydrogen bonds between dA-dT and dG-dC base pairs in A, B, or Z motifs. A few duplex structures have local distortions in the double helix, such as bending, twisting, and unwinding, leading to the formation of kinks or loops. Also included are guanine-rich DNA quadruplexes, with regions where four guanine bases interact through hydrogen bonding to create a four-stranded configuration.
2. **RNA fragments**. A set of 29 RNA structures (Table S2) were studied, comprising 638 A, 679 C, 865 G, and 616 U residues. The data set comprises RNA molecules with varied tertiary structures and functionality. It contains mRNA, tRNA, rRNA, ribozymes, and riboswitches. Other types of RNA are also present, like snoRNA, crRNA, xrRNA, and aptamers. Some synthetic structures, such as a paranemic crossover RNA triangle, are also included in the data set. Most of these structures are single-stranded RNA molecules, folding into complex quaternary structures.
3. **DNA–protein complex**. We investigated the binding interaction between a transcription factor protein and a 25-base-pair DNA oligomer, which plays a key role in the post-translational modification of histones (Table S3). Specifically, we focused on the Nuclear Transcription Factor Y (NF-Y) and its complexation with the DNA sugar–phosphate backbone (PDB ID: 4AWL). Our objective was to analyze the hydropathy of the DNA–protein complex and the individual DNA and protein components to elucidate the role of hydropathy in facilitating the formation of this complex.
4. **RNA**–**protein complexes**. We analyzed the structure of the RNA–protein complex (Table S3), focusing on two examples: a small nuclear RNA bound to a protein (PDB ID: 5TF6) and a 104-nucleotide RNA fragment interacting with the ribosomal proteins S15, S6, and S18 (PDB ID: 1G1X). For both structures, we assessed the PARCH values of the amino acid residues interacting with the nucleotides within the complexes and as individual components to understand the role of hydropathy on the molecular-level interactions.

### Approach

#### System Preparation and Optimization

The structures used in this study (Tables S1 and S3) were obtained from the Protein Data Bank. For each case, the target molecules—such as DNA, RNA, or protein—were isolated, and all extraneous chemical species were removed. The simulations were performed using Gromacs 2023.2 ^39^ and CHARMM36 force field.^40^ In a separate set of simulations, DNA molecules were also modeled using the other available force fields—OL15 and BSC1.^41,42^ The water molecules were modeled using the TIP3P^43^ parameters. Each structure underwent energy minimization and equilibration prior to the production simulation runs. To prepare the system for simulation, the target molecule was placed at the center of a cubic simulation box, ensuring a minimum distance of 1.5 nm between the molecule’s surface and the box edges in the *x*, *y*, and *z* directions. The molecule was then solvated in a 0.15 M NaCl solution, with counterions added to neutralize the system’s overall charge. The system was energy minimized using the steepest descent algorithm,^44^ followed by a short 2 ns equilibration run under isothermal-choric (NVT) condition, where the molecule was position restrained. This was followed by unrestrained equilibration using the isothermal-baric (NPT) condition, run for 2 ns at 300 K. The pressure of the system was maintained at 1 bar using Berendsen barostat^45^ with a compressibility constant of 4.5 × 10^–5^ bar^–1^. During the NPT and NVT steps, the temperature was controlled at 300 K using the v-rescale thermostat.^46^ A subsequent 10 ns production run was conducted under NPT conditions at 300 K and 1 bar, with the pressure regulated using the Parrinello–Rahman barostat^47^ and the same compressibility constant. The Linear Constraint Solver (LINCS)^48^ algorithm was employed to constrain bonds involving hydrogen atoms for all three stages. Long-range electrostatic interactions were computed using the particle-mesh Ewald (PME)^49^ method with a Fourier grid spacing of 0.12 nm and cutoff distances of 1.0 nm for short-range neighbor list and 1.2 nm for van der Waals interactions. The MD was performed for 10 ns by using the simulation parameters in the previous NPT equilibration step.

#### PARCH Workflow

The final frame from the MD production equilibration step was selected for the PARCH analysis. The optimized molecule was stripped of all water and rehydrated with a 4.5 Å water shell, which is specific to nucleic acids. This thickness was determined based on the radial distribution function (RDF) between the phosphorus atoms of the nucleic acid backbone and the oxygen atoms of the water solvent (Figure S1), which is slightly larger than the 4.15 Å shell used for proteins. The parameters utilized for PARCH calculations are listed in Table S4.

To ensure system neutrality, hydrated counterions were added at a minimum distance of 3 Å from the molecular surface and from each other to prevent undesired interactions. The boundaries of the simulation box were set to 3 Å beyond the outer edge of the water shell. The system underwent simulated annealing from 300 to 800 K at a controlled heating rate of 1 K/10 ps in an NVT ensemble, with periodic boundary conditions. The annealing production simulations were performed in triplicate, and position restraints with a force constant of 10,000 kJ mol^–1^ nm^–2^ were applied to the molecule and ions to prevent conformational changes in the target structure.

During the 5 ns annealing process, the number of water molecules within a 4.5 Å cutoff of each nucleotide was recorded. Two separate values were recorded for each residue: one for the BB and one for the NB. For proteins, the number of water molecules was calculated at a 3.15 Å cutoff around each amino acid. We performed the PARCH calculations using our own Python code. The computed PARCH values were added to the B-factor column of the PDB files. These modified PDB files, annotated with hydropathy data, can be easily visualized in software suites, enabling an intuitive interpretation of the hydropathy distributions.

## Results and Discussion

Here we show that PARCH analysis captures how the three-dimensional structure and local nucleotide geometry of DNA and RNA critically shape their hydropathy and biophysical properties.

### The DNA Backbone Is More Hydrophilic Than the Bases

DNA backbone exhibits an average PARCH value of 4.28 ± 1.39, in stark contrast to the bases with an average PARCH value of 0.57 ± 0.68. This order-of-magnitude difference is largely due to the distinct chemical properties of the backbone and the bases. The PARCH value probability density distributions further highlight this striking difference, illustrating the broad range of values spanned by the backbone compared to the bases (Figure 2). Specifically, the backbone PARCH value distribution is right-skewed with values ranging from 1.4 to 13.7. Higher PARCH values are commonly observed in regions where the DNA chain bends or in complex secondary structures, namely, quadruplexes, while lower values are sometimes seen at the chain ends. The attached bases dA, dT, dG, and dC slightly influence the PARCH values of the backbone with average values of 3.9, 4.6, 4.3, and 4.3, respectively. In contrast, the PARCH values of the bases range between 0.03 and 6.1. The average PARCH values for dA, dT, dG, and dC are 0.4, 0.7, 0.6, and 0.4, respectively. Some outliers exist for the bases due to the flipping of residues in single-stranded DNA structures.

**Figure 2:**
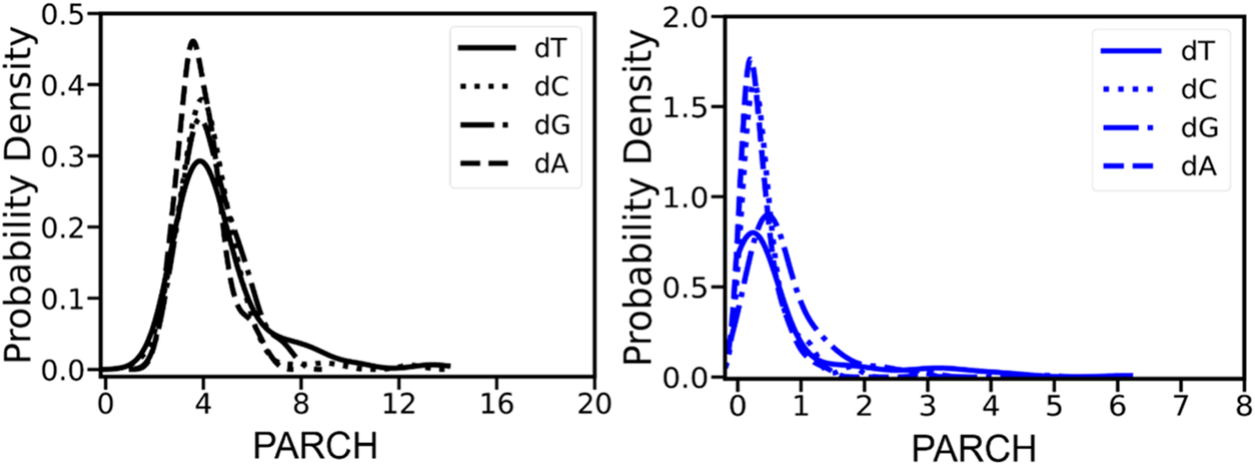
Probability distribution of PARCH values of DNA nucleotides. The left panel displays the probability distribution of PARCH values for the backbone (black), while the right panel shows the distribution for the bases (blue) for better comparison. The backbone PARCH values cover a wider range, with the highest probability occurring around a PARCH value of 4. In contrast, the PARCH values of the bases span a narrower range, with the highest probability centered around a PARCH value of 0.5.

To determine if there was any significant difference in the PARCH values due to the difference in the chemical structure of the four bases, we computed the PARCH value distributions of all DNA nucleotides (139 dA, 132 dC, 180 dG, and 146 dT) in our data set. We used the Kruskal–Wallis test for the backbone and found that dA in our database was significantly different from dT and dG, while other residues have no significant differences (Figure S2). Interestingly, PARCH value distributions for the bases show that dG is significantly different from all other nucleotides (Figure S2). This difference can be attributed to a higher number of dG in our data set that includes G-quadruplexes.

### Structural Variations in DNA Duplexes Impact the Local Hydropathy of Nucleotides

A detailed examination of DNA duplexes highlights the significant impact of base pairing on hydropathy. To explore these effects, we analyzed multiple duplex structures and present three illustrative examples featuring normal, flipped, and unpaired base pairs (Figure 3).

**Figure 3:**
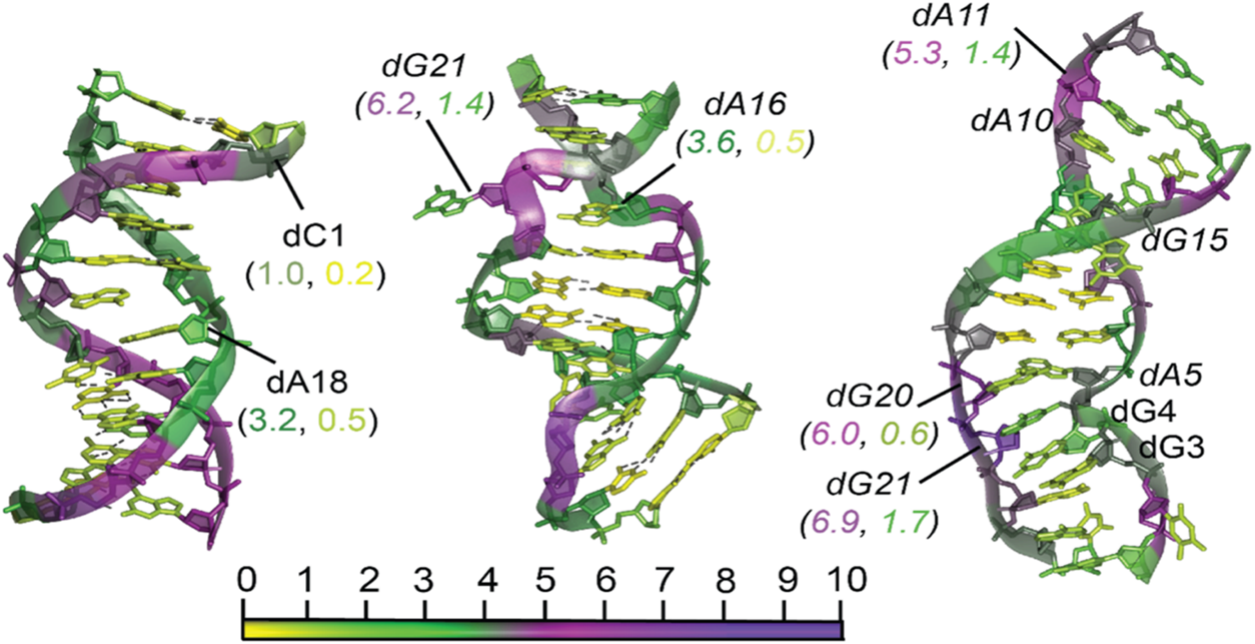
Effect of the local DNA structure on the hydropathy of nucleotides. The image illustrates three types of DNA duplexes: standard (PDB ID: 6X5D, left), flipped base (PDB ID: 111D, middle), and unpaired bases (PDB ID: 103D, right). Each structure was subjected to a 10 ns MD simulation before PARCH analysis. The residues are color-coded according to the PARCH scale at the bottom for visual reference. The PARCH values for the backbone and nitrogenous bases are labeled in (BB, NB) format. Residues discussed in the text are shown in italics for easier identification.

The first duplex, a 24-residue structure (PDB ID: 6X5D),^50^ consists of two antiparallel DNA strands that form a classic B-DNA motif. The strands are stabilized by hydrogen bonds between the dA-dT and dG-dC base pairs. In this normal duplex, the terminal residues exhibit lower PARCH values compared to the rest of the structure (Figure 3). The grooves of the duplex display considerably higher PARCH values, reaching up to 5, while the remainder of the backbone has moderately high values around 4. The nitrogenous bases maintain relatively low PARCH values, rarely exceeding 1, with an average of about 0.6.

In contrast, the second duplex (PDB ID: 111D)^51^ is a synthetic dodecanucleotide, d­(CGCAAATTGGCG), which forms a B-DNA helix with ten standard Watson–Crick base pairs and two dA-dG mismatches. These mismatches disrupt the standard hydrogen bond formation between the bases, leading to localized structural distortions. In one instance, an adenine base flipped, significantly increasing the PARCH value. The flipped residue, dG21, in the middle duplex exhibits significantly higher PARCH values (6.2, 1.4), reflecting its increased hydrophilicity and exposure. However, its complementary inner residue dA16 remains in a standard configuration and displays standard PARCH values (3.6, 0.5). The backbone surrounding these disrupted base pairs also becomes highly hydrophilic, with the highest PARCH values observed in this region (Figure 3).

The third duplex (PDB ID: 103D)^52^ is an unusual example because it has four unpaired guanosine nucleotides and four mismatched adenosine-guanosine base pairs, resulting in a distorted backbone (Figure 3). Regions near the unpaired dG residues exhibit lower-than-average PARCH values in both the backbone and base regions. However, one unpaired guanosine residue, dG21, shows elevated PARCH values for both backbone and base (6.9, 1.7), likely due to a bulge in the backbone at this position. Three of the four mismatched dA-dG pairs remain in a standard configuration without flipping and exhibit average PARCH values (Figure 3). However, the fourth pair, dG20-dA5, deviates from the plane, leading to an increased PARCH value for the G20 residue (6.0, 0.6). Notably, the region surrounding one such pair, dA10-dG15, shows increased hydrophilicity around the backbone of dA11 (5.3, 1.4). While this observation suggests a possible influence of the base mismatch, the connection between the increased hydrophilicity and the mismatch remains inconclusive.

These examples highlight the sensitivity of hydropathy to structural disruptions within DNA duplexes and provide insight into how mismatched and flipped bases contribute to changes in hydration and molecular interactions.

### Asymmetry in DNA Quadruplex Strands Leads to Differences in Hydropathy of Nucleotides

We performed PARCH analysis of the *B-raf* DNA quadruplex,^53^ which consists of two strands of the 20-mer sequence d­(GGGCGGGGAGGGGGAAGGGA). These strands, labeled 1 and 2, intertwine to form an asymmetric structure (inset in Figure 4) stabilized by a core of six vertically stacked potassium ions. Each potassium ion is coordinated to coplanar dG residues, creating a stack of seven consecutive dG-quartets. Figure 4 provides side and top views of the quadruplex structure. The hydropathy analysis revealed several key observations:

**Figure 4:**
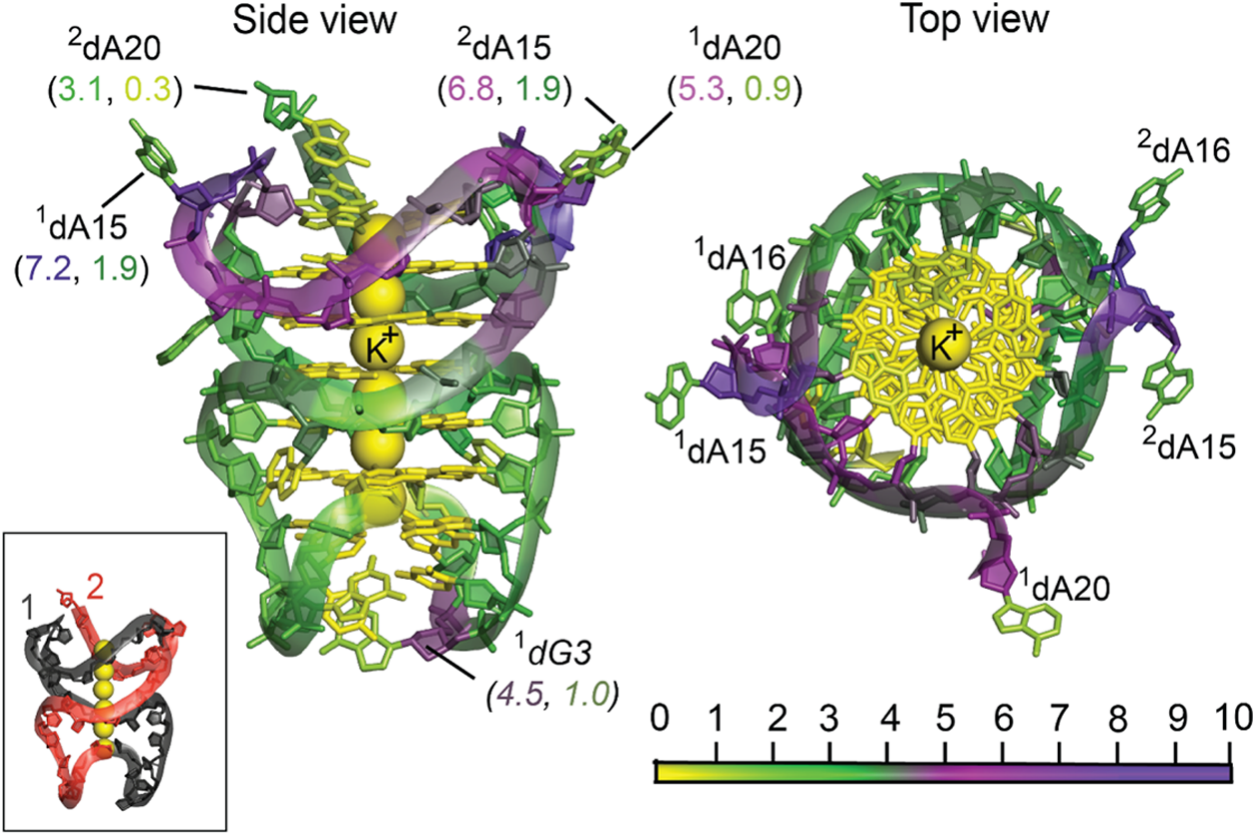
Hydropathy of DNA G-quadruplex (PDB ID: 4H29). The inset (bottom left) shows the B-raf DNA quadruplex, which consists of two strands of the 20-mer sequence in gray (labeled 1) and red (labeled 2), intertwining to form an asymmetric structure and are stabilized by a core of six vertically stacked potassium ions.  The left panel shows the side view of the quadruplex with G-tetrads around the potassium core. The right panel shows the quadruplex from the top, highlighting the planar arrangement of the G-tetrads. Specific residues are labeled to emphasize hydropathy variations: hydrophilic regions are prominent in the loop regions, while the dG stack and potassium core are predominantly hydrophobic. The structure was subjected to a 10 ns MD simulation before PARCH analysis. Nucleotides are color-coded based on PARCH values, as indicated by the scale at the bottom. The PARCH values for the backbone and nitrogenous bases are labeled in (BB, NB) format.

- *Conformational Differences and Hydrophilicity*: Despite the sequence similarity between the two strands, the PARCH values of their residues differ, attributable to conformational variations. On average, strand 1 has more hydrophilic residues than strand 2. This difference may be due to sharp conformational kinks in the strand 1 backbone at residues ^1^dG3, ^1^dG14, ^1^dA15, and ^1^dG17.
- *Hydrogen Bonding and Low PARCH Values*: Most dG residues have low PARCH values because they participate in hydrogen bonding within the strand or with complementary residues in the opposing strand. This observation is particularly true for the dG residues involved in quartets with potassium ions, leading to low PARCH values in the potassium core.
- *Flipped Residues and Water Accessibility*: In contrast, flipped residues such as ^1^A15, ^1^A20, ^2^A15, and ^2^A20 exhibit higher water accessibility, resulting in elevated PARCH values.
- *Asymmetry and the G·C·G·C Quartet*: Asymmetry and the lack of hydrogen bonding among residues in the G·C·G·C quartet at the bottom of the quadruplex contribute to a higher PARCH value for ^1^dG3 (4.5, 1.0). The three other residues in the quartet—^1^dC4, ^2^dG3, and ^2^dC—are also twisted out of plane. However, their proximity to the quartet core results in comparatively lower PARCH values.

This analysis highlights the structural and hydropathic complexity of the *B-raf* DNA quadruplex, emphasizing how subtle conformational changes influence the hydropathy of the molecule.

To further validate the hydropathy trends observed in DNA duplexes and quadruplexes, we analyzed the PARCH values of a synthetic DNA molecule (PDB ID: 8DUT),^54^ which contains both duplex and quadruplex regions (Figure 5). The duplex regions display well-ordered helical structures, with backbone PARCH values ranging from 4 to 8. For example, residues such as dA57 (2.5, 0.1) and dT83 (4.4, 0.4) exhibit relatively low PARCH values, consistent with the structured nature of the duplex. In contrast, the quadruplex region, which is rich in dT and dG nucleotides, features instances of base flipping that result in significantly elevated PARCH values, often exceeding 8. Notable examples include dT22 (12.9, 3.2), dT26 (7.1, 4.4), and dT30 (10.0, 2.8). These elevated values highlight the increased accessibility of flipped bases to water and the less-ordered conformation of the quadruplex region.

**Figure 5:**
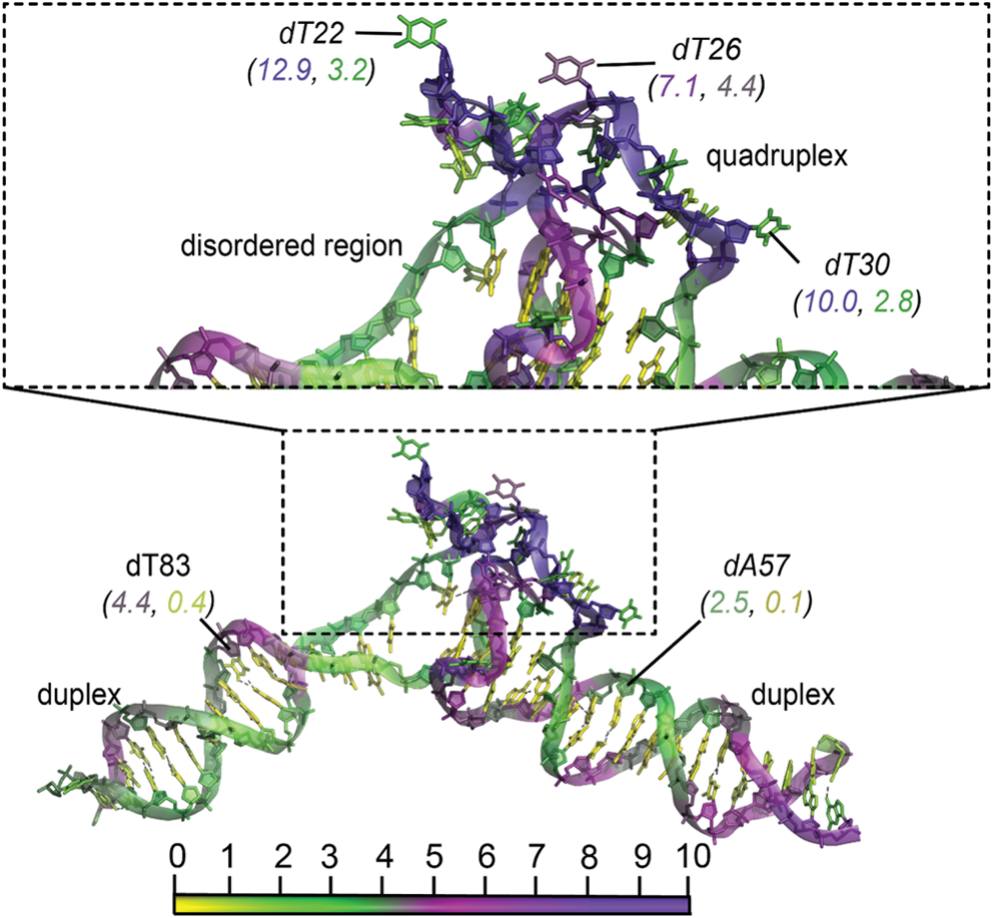
Hydropathy of a duplex-quadruplex complex (PDB ID: 8DUT). The lower panel shows the entire DNA molecule with two ordered duplex motifs, a disordered region, and a central quadruplex region. All nucleotides are colored according to the PARCH values (bottom). The upper panels provide a zoomed-in view of the disordered region lacking base pairing, highlighting residues dT22, dT26, and dT30. These residues exhibit higher values in both backbone and base regions compared to the more stable, ordered duplex motifs (for example, residues dA57 and dT83), reflecting the increased hydropathy associated with structural disorder. Nucleotides are color-coded based on PARCH values, as indicated by the scale at the bottom. The structure was subjected to a 10 ns MD simulation before the PARCH analysis. The PARCH values for the backbone and nitrogenous bases are labeled in (BB, NB) format. Residues discussed in the text are shown in italics for easier identification.

These findings emphasize the stark hydropathy variations between the organized, stable duplex regions and the dynamic, less-ordered quadruplex regions, driven by differences in the structure and base interactions.

### The Structure Complexity of RNA Makes It More Hydrophilic Than DNA

Our work shows that RNA, which forms structures more complex than DNA, is more hydrophilic. This attribute is primarily due to its single-stranded nature and the chemical differences between RNA and DNA. Unlike the double-stranded, stable DNA helix, RNA is usually single-stranded, which allows it to fold back on itself and form intricate secondary and tertiary structures through intramolecular base pairing.

These structures, such as hairpins, loops, bulges, and pseudoknots, have higher affinity for water, as the tertiary structures are less compact and give RNA the ability to form hydrogen bonds between complementary bases within the same strand.

The 2′-hydroxyl group in the ribose sugar of RNA enables additional hydrogen bonding, allowing RNA to adopt diverse and complex three-dimensional structures. These complex structures are crucial for RNA’s diverse functions, such as catalysis (ribozymes), structural roles (rRNA), and regulatory activities (microRNAs and long noncoding RNAs). In contrast, DNA’s primary role as a stable, double-stranded genetic information carrier limits its structural complexity. The data set consists of different types of RNA molecules, namely, mRNA (mRNA), tRNA (tRNA), rRNA (rRNA), precursor microRNA (pre-miRNA), ribozyme, and riboswitch.

The hydropathy of the RNA backbone ranges from 0.03 to 19.9 (Figure 6). The average PARCH values of the backbones of the A, U, G, and C residues are 4.9, 4.8, 5.3, and 4.8, respectively. The mode PARCH value is 4.5. Like DNA, the backbone PARCH values are an order of magnitude higher than the bases. Figure 6 shows the PARCH range for the bases; the values vary from 0 to 8.1. Although the PARCH values of the four bases A, U, G, and C are 0.4, 0.4, 0.4, and 0.3, respectively, higher values are observed in complex structure geometries with high residue densities often resulting in distortions and base flipping.

**Figure 6:**
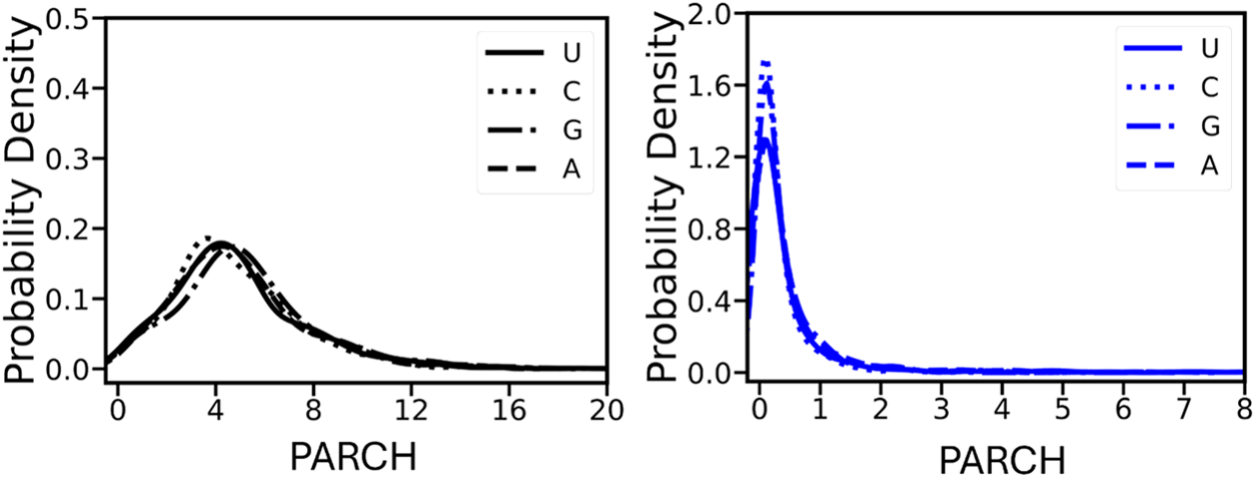
Probability distribution of PARCH values of RNA nucleotides. The left panel displays the probability distribution of PARCH values for the backbone (black), while the right panel shows the distribution for the bases (blue) for better comparison. The backbone PARCH values cover a wider range, with the highest probability occurring around a PARCH value of 4.5. In contrast, the PARCH values of the bases span a narrower range, with the highest probability centered around a PARCH value of 0.4.

To determine if there was any significant difference in the PARCH values due to the difference in the chemical structure of the four bases, we computed the PARCH value distributions of all RNA nucleotides—638 A, 679 C, 865 G, and 616 U—in our data set. We used the Kruskal–Wallis test for the backbone and found that G was significantly different from all of the other residues. None of the other nucleotide pairs have significant differences in the backbone PARCH values (Figure S3). Interestingly, PARCH value distributions for base pairs A and C show significant differences, but it is not clear what caused this difference.

### PARCH Analysis of RNA Structures Reveals Intricate Relationship between Structure and Hydropathy

We analyzed the PARCH values across various types of RNA structures, highlighting key observations for each category using representative example structures.

The **mRNA** acts as a template for protein translation by carrying genetic information from DNA to the ribosome, where proteins are synthesized. A specific region of mRNA, known as the untranslated region (UTR), frequently contains G-quadruplexes, which play critical roles in regulating gene expression. In this study, we analyzed one such G-quadruplex structure (PDB ID: 7SXP),^55^ a single-stranded, 22-nucleotide mRNA sequence 5′(UGUGGGAUGGGCGGGUCUGGGA)­3′ with a G8U substitution. The underlined guanine residues form a 4-fold symmetric, coplanar arrangement around core potassium ions, resulting in low PARCH values. These guanine residues are also stabilized by hydrogen bonding, which reduces their interaction with the surrounding water molecules. In contrast, the loops surrounding the G-quadruplex, particularly at positions C12 and C17, are twisted out of plane and exhibit increased hydrophilicity compared to the rest of the molecule (Figure 7). This structural distinction highlights the balance between the hydrophobic core of the G-quadruplex and the hydrophilic nature of its surrounding loops, underscoring its role in mRNA function and gene regulation.

**Figure 7:**
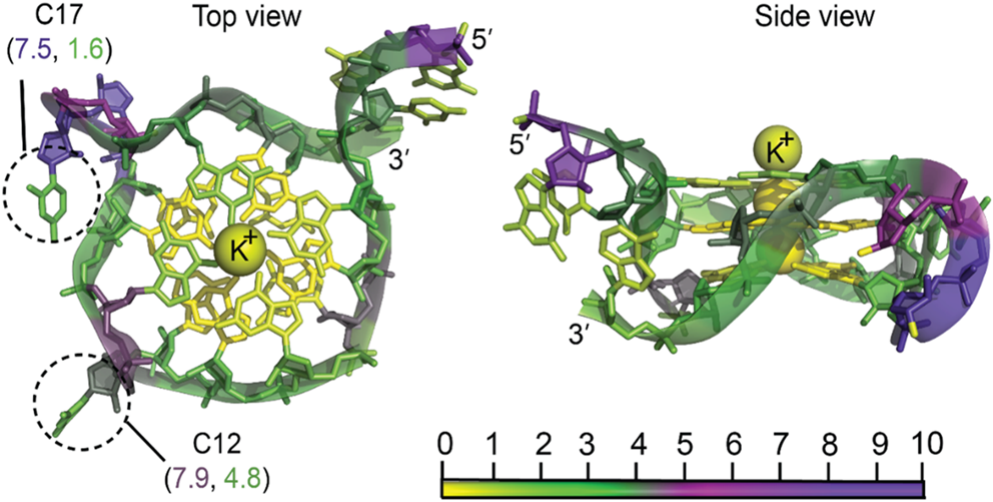
Hydropathy analysis of G-quadruplex of untranslated region (UTR) of mRNA (PDB ID: 7SXP). Nucleotides are color-coded according to the PARCH scale (bottom). The top view (left) highlights the planar arrangement of G-tetrads around the potassium ion core, and the side view (right) illustrates the stacking of G-tetrads. Labeled residues (C12 and C17) emphasize the hydrophilicity of the flipped loops. The structure was subjected to a 10 ns MD simulation before the PARCH analysis. The PARCH values for the backbone and nitrogenous bases are labeled in (BB, NB) format.

The **tRNA** plays a crucial role in the splicing of pre-mRNA, an essential step in mRNA processing before translation. We computed the PARCH values for tRNA (PDB ID: 7KJU)^56^ bound to KEOPS (Kinase, Endopeptidase, and Other Proteins of Small size), a highly conserved five-subunit protein complex critical for cellular fitness and animal development. Hydropathy analysis of tRNA provides valuable insights that align with experimental findings.

The tRNA is known to bind the protein in its 3′ region, specifically via residues G73, C74, and C75. The backbone PARCH values in this region are approximately 4 (Figure S4), indicating moderate hydrophilicity. Experimental evidence suggests that the bases of these residues, rather than the backbone, interact directly with the protein. These bases exhibit PARCH values between 0.1 and 0.2, comparable to those of hydrophobic amino acids. Residues C73 and C75 interact with the Ile and Gln residues of the KEOPS protein. Additionally, residues G68 and G69, which bind to Arg residues in KEOPS, also have backbone PARCH values near 4. In contrast, the backbone of residues C10–U11 is predicted to make repulsive interactions with a Glu residue in KEOPS, resulting in higher PARCH values of approximately 6.

The **rRNA** is a key structural and functional component of ribosomes, facilitating mRNA translation into the protein. We computed PARCH values for a 16S subunit of rRNA in . (PDB ID: 1K5I).^57^ The structure has a hairpin loop with a C-A base pair mismatch, causing the loop to have the highest PARCH value of the molecule (Figure S5). The remaining structure forms an A-form helix, and like the DNA duplex, the grooves in the structure have higher PARCH values.

The **riboswitches** are predominantly located in the 5′ UTR of bacterial mRNA, where they regulate gene expression. In this study, we analyzed the 52-nucleotide RNA structure of the *Candidatus koribacter versatilis* riboswitch domain 1 (PDB ID: 6TB7),^58^ which binds to NAD^+^. For the hydropathy calculations, the NAD^+^ molecule was removed to compute the PARCH values of the nucleotides. Structurally, the riboswitch consists of a helical domain and an eight-nucleotide bulge formed by the residue sequence A^8^C^9^A^10^A^11^C^12^C^13^C^14^C^15^ (Figure 8). The backbone regions of A8 to C14 exhibit high PARCH values due to sharp kinks introduced by the bulge. However, the bases of C13 and C14 form hydrogen bonds with G36 and G38 in a hairpin-bulge motif, resulting in low PARCH values of 0.30 and 0.31, respectively. The residue following the bulge, G16, interacts in-plane with C45 through hydrogen bonding, which also lowers its PARCH values. In contrast, the NAD^+^ binding site, comprising residues A8, C9, and A10, is characterized by elevated PARCH values, reflecting its high hydrophilicity and accessibility (Figure 8). These observations highlight the intricate hydropathic variations associated with the structural and functional features of the riboswitch.

**Figure 8:**
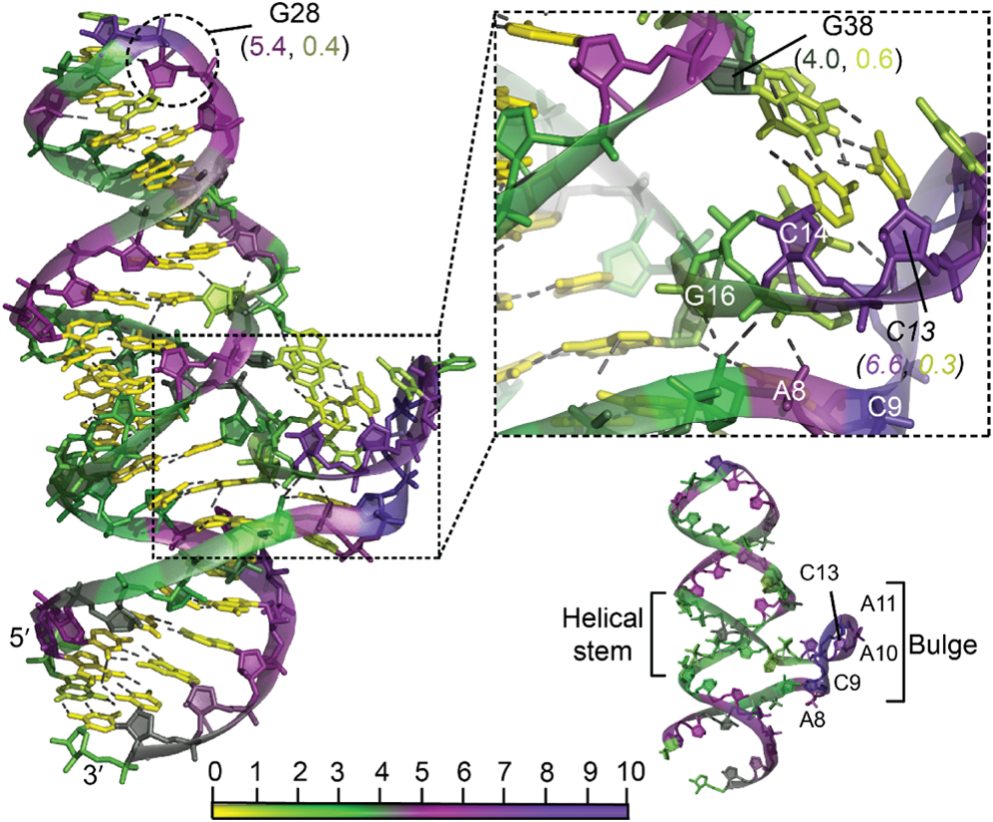
Hydropathy of riboswitch (PDB ID: 6TB7) reveals a bulged stem-loop motif. The zoomed-in inset shows the bulge formed by residues A^8^C^9^A^10^A^11^C^12^C^13^C^14^C^15^, which interact with the stem-loop through two base pairings—C^13^:G^36^ and C^14^:G^38^. The bottom-right structure presents a simplified view of the bulge-stem motif, excluding base pairings for the sake of clarity. All nucleotides are color-coded based on the PARCH scale (bottom). The structure was subjected to a 10 ns MD simulation before the PARCH analysis. The PARCH values for the backbone and nitrogenous bases are labeled in (BB, NB) format.

A **ribozyme** is a type of RNA molecule capable of catalyzing specific biochemical reactions, such as the function of protein enzymes. Here, we analyzed the PARCH values of a recent cryogenic electron microscopy (cryo-EM) ribozyme structure (PDB ID: 7YC8)^59^ during the first step of its self-splicing process. The structure was obtained from *Tetrahymena*, a unicellular ciliated eukaryote that lives in freshwater. Due to a complex conformation, we see various secondary structures emerge, including duplexes and quadruplexes. High PARCH value is observed on the backbone regions of the residues that lie on the outer edge of the molecule. The molecules inside, however, exhibit a lower PARCH value. Guanosine triphosphate (GTP) binds to the residues in the core of the molecule, namely, G264, A265, C266, C311, G312, and G313 (Figure 9). Although surrounded by high PARCH value for backbone, these residues exhibit a lower PARCH value. An unusual occurrence of the higher PARCH value of the base compared to the PARCH value of the backbone is seen for G313 (0.6,0.9). Experimental studies show that the purine of GTP interacts with these residues. The first few starting 5′ residues consequently interact with the triphosphate group of the GTP to participate in splicing, and these residues show a high PARCH value, despite terminal residues usually being hydrophobic. Another instance of base PARCH value greater than the backbone is observed for the terminal residue G22 (5.2, 5.8) at the 5′ end. This ribozyme structure has a high water retention capability due to the strong negative charge density that surrounds the molecule, which allows PARCH value to shoot up to as high as 17.0.

**Figure 9:**
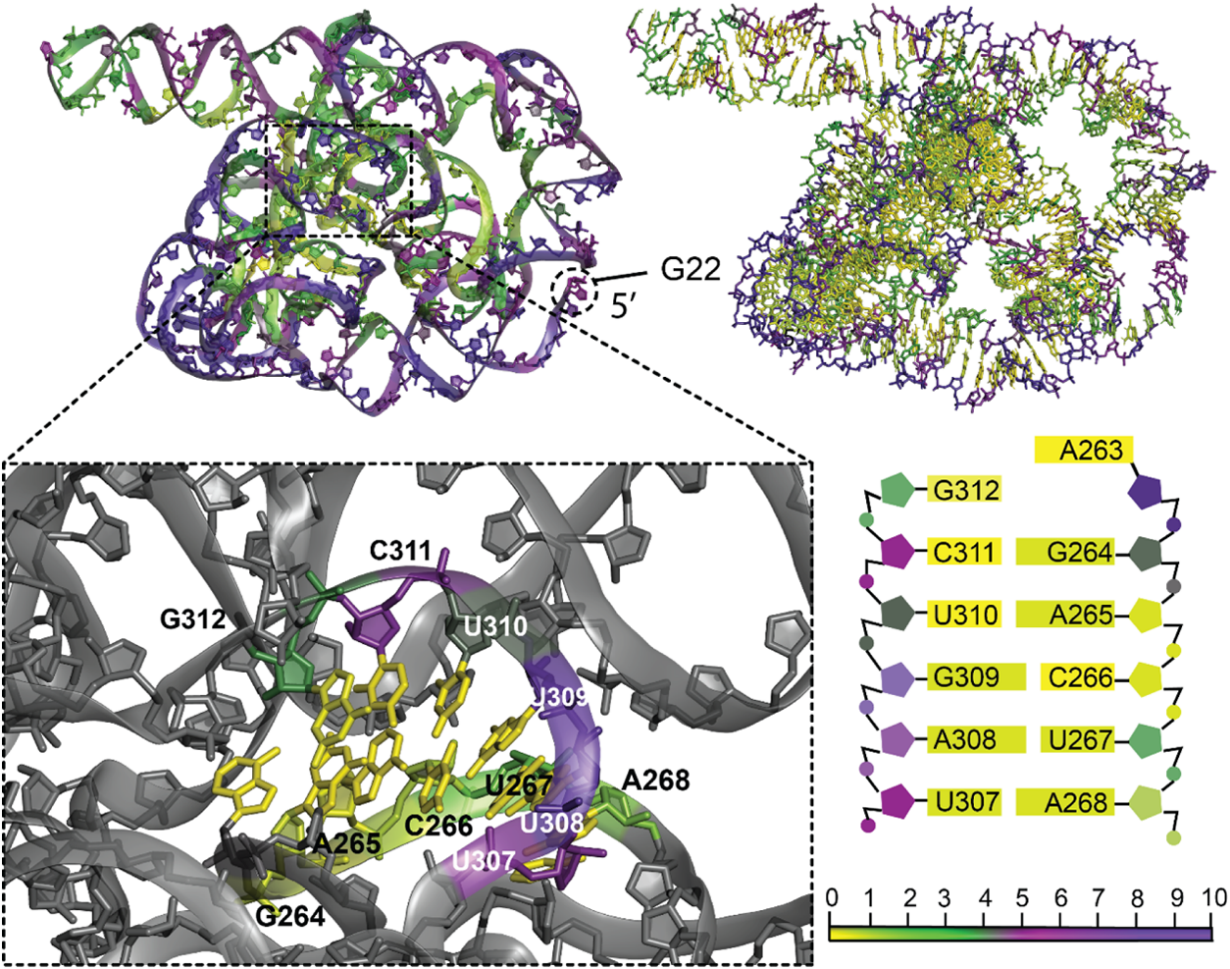
Hydropathy of the ribozyme (PDB ID: 7YC8) during the first step of its self-splicing process. The top-left panel illustrates the backbone hydropathy values using the PARCH scale, while the top-right panel emphasizes base pair interactions within the 388-residue ribozyme. A zoomed-in view highlights the binding site along with the critical interactions between core nucleotides (264–268 and 307–312) and the guanosine triphosphate (GTP); noncore residues are grayed out for clarity. The bottom-right panel provides a detailed schematic mapping of specific nucleotide contacts and their structural roles in the ribozyme core. Nucleotides are color-coded based on the PARCH scale (bottom). The structure was subjected to a 10 ns MD simulation before the PARCH analysis. The PARCH values for the backbone and nitrogenous bases are labeled in (BB, NB) format.

### PARCH Provides a Unified Hydropathy Framework for Nucleic Acid–Protein Complexes

We used the PARCH scale calculations to investigate the hydropathy of the nucleic acid and protein interface using one example of DNA–protein and two for the RNA–protein complex. For clarity in this article, amino acids are denoted by their three-letter codes, and their PARCH values are reported as single numbers in parentheses.

The DNA–protein complex (PDB ID: 4AWL)^60^ shows the binding of Nuclear Transcription Factor Y subunit (NF-Y) to DNA oligonucleotide. The DNA residues that contact the protein residues have PARCH values lower than those of the rest of the DNA chains (Figure 10). For example, ^2^dG-22 and ^2^dA-21 have backbone PARCH values of 2.3 and 2.9, respectively. The surrounding DNA residues, however, have higher BB PARCH values, ranging from 5.19 to 6.39. The base PARCH values of these residues are much lower, rarely going above 1.5. Similarly, dC16 has a backbone PARCH value of 1.64, ^2^dT-11 has a backbone PARCH value of 0.6, and residues ^1^8–12 comprising the DNA oligonucleotide also show lower backbone PARCH values, ranging from 1.95 to 3.26, except for ^1^dT12, which is 5.1. These values show that upon contact with protein, the PARCH values of these residues decrease, indicating strong electrostatic binding. Notably, many of the protein contacts are with Lys and Arg residues, which would suggest an electrostatic interaction as well. These contact residues corroborate those mentioned in the literature.

**Figure 10:**
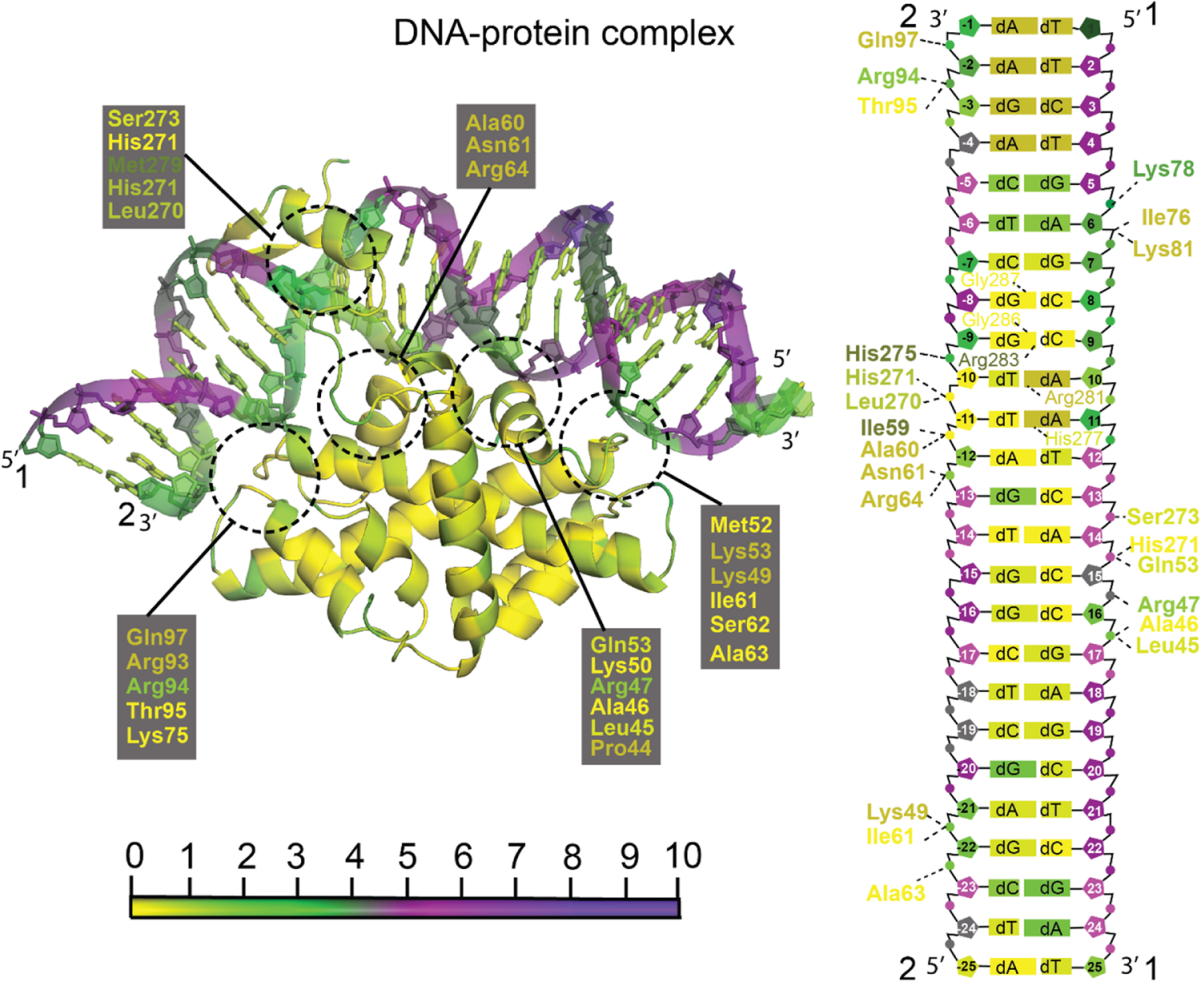
PARCH analysis of the DNA–protein complex (PDB ID: 4AWL). The 3D structure (left) illustrates the DNA oligonucleotide bound to the Nuclear Transcription Factor Y subunit (NF-Y), highlighting key interaction regions with dashed circles. Residues are color-coded according to the PARCH scale (bottom). The right panel provides a detailed schematic mapping of specific contacts with dashed lines linking amino acid residues to either the DNA backbone phosphates or the bases. The 5′ and 3′ ends of the DNA strands are labeled in the cartoon representation to enable a clear correspondence between the 3D structure and the 2D schematic. The complex was subjected to a 10 ns MD simulation before PARCH analysis. The PARCH values for the backbone and nitrogenous bases are labeled in (BB, NB) format.

The PARCH analysis has also been on the cleaved protein to compare the PARCH values of the protein in complex with the cleaved protein. Upon comparison, we see that the contact residues of the protein are more hydrophilic in the cleaved protein. The overall cleaved protein, apart from contact residues, also shows a more hydrophilic nature. Some of the contact residues in the cleaved protein and complex are highlighted in the Supporting Information (Figure S6). The PARCH analysis was also performed on the cleaved DNA, but significant results were not obtained from that analysis. DNA also would not be found freely physiologically, thus not obscuring our understanding of complex behavior.

Further, we examined the hydropathy of a small nuclear ribonucleoprotein (snRNP) (PDB ID: 5FT6)^61^ using the PARCH scale. It shows the binding of the highly conserved U6 snRNA with Prp24 in a unique interlocked topology. We note that the RNA residues, A40, A46-G52, U54, A56, and C61—in contact with the protein have lower PARCH values. These residues have backbone PARCH values less than 4, while the noncontacting RNA residue backbones have higher PARCH values in the 5–6 range. When we computed the PARCH values of the protein without the RNA contact, the protein residues have much higher PARCH values, indicating that the binding of protein is electrostatic interactions (Figure 11). The PARCH analysis of the RNA molecule did not show a significant change in its PARCH values without the protein (Figure S7). In essence, the remarkable ability of DNA and RNA to fold into a diverse array of structures grants them a wide range of hydropathic properties, which are fundamental to their multifunctional roles within the cell.

**Figure 11:**
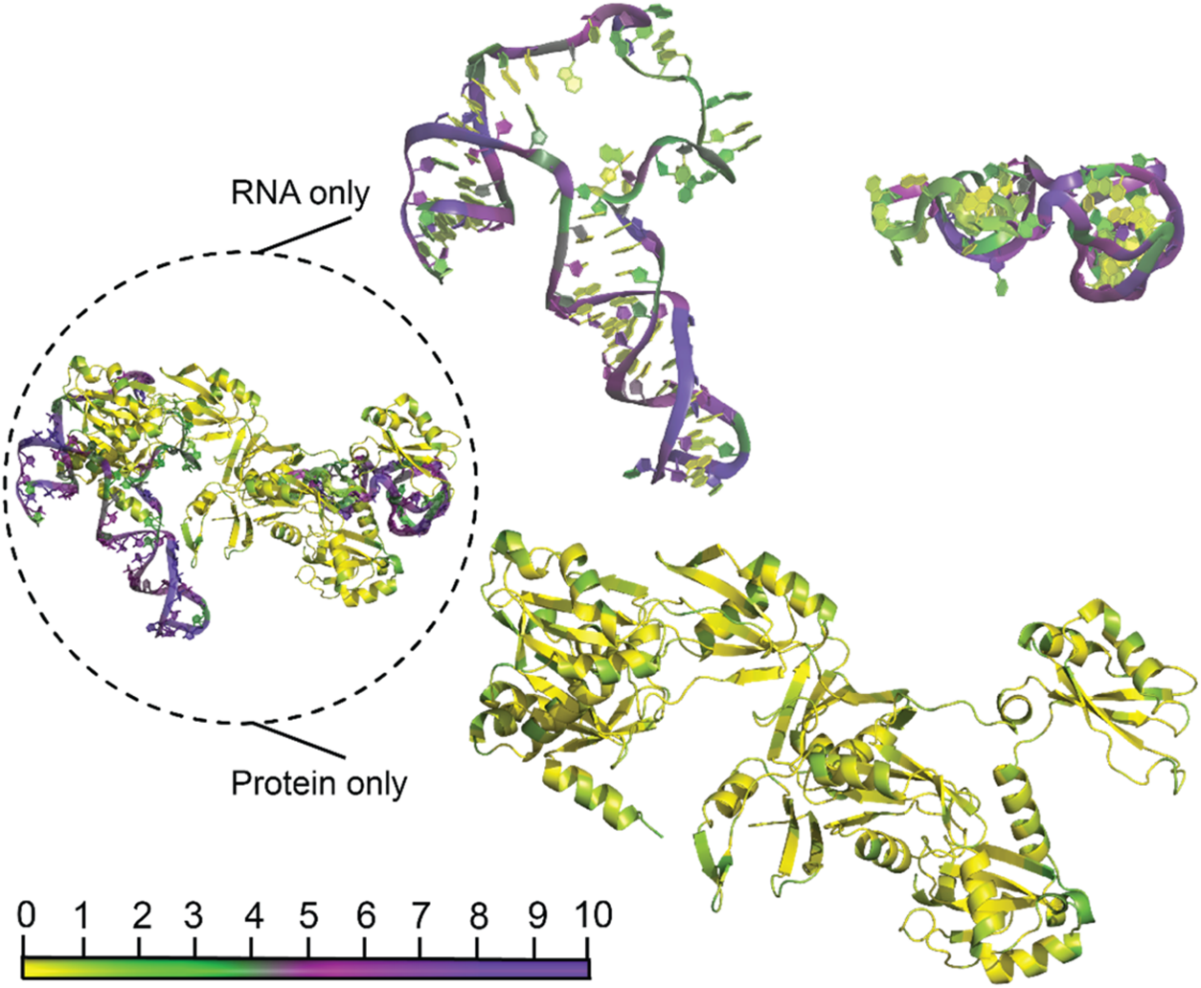
PARCH analysis of the RNA–protein complex (PDB ID: 5TF6). The figure showcases the RNA–protein complex (in dashed circle) and RNA-only (top), and protein-only (bottom right) structures. The RNA-only (top) has two symmetric nucleotide fragments that are rotated out of plane from each other but bind to the protein (bottom) to form the complex. The base pairs are not shown in the RNA-only structure for clarity. All residues are color-coded according to the PARCH scale (bottom left), indicating interaction properties and structural features. The complex was subjected to a 10 ns MD simulation before the PARCH analysis.

Notably, RNA’s structural flexibility enables it to go beyond the conventional function of merely preserving genetic information. Instead, RNA serves as a crucial participant in catalysis, regulation, and coordination of intricate cellular processes.

### PARCH Values Reveal Hydropathy Patterns in Nucleic Acids

Our PARCH analysis data show that the sugar–phosphate of the backbone is significantly more hydrophilic than the bases in both DNA and RNA molecules. This observation is a fundamental property of nucleic acids that allows these molecules to adapt to their ionic environments and support essential biological functions. The interaction between the backbone and water molecules helps stabilize the overall structure of the nucleic acids. In contrast, the relatively hydrophobic nature of the bases drives their stacking, which forms the core of the DNA double helix or folded RNA structures.

Our findings confirm that standard DNA motifs, such as double-stranded duplexes, align with the established hydropathy pattern: a highly hydrophilic (high PARCH value) backbone and low hydropathy values for the bases. However, structural distortions in the nucleotide sequence, such as bulges, base flips, or mismatches in the double-stranded DNA helix, result in increased hydropathy for both the backbone and bases (Figure 12). These elevated PARCH values significantly impact how DNA interacts with proteins, influencing critical processes like transcription and replication. Additionally, specialized motifs such as G-quadruplexes exhibit high PARCH values due to the stacking of planar guanine tetrads (Figures 4 and 5), which are stabilized by hydrogen bonds. The hydrophilicity of these DNA G-quadruplexes plays a crucial role in regulating cellular processes, making them a key focus for both fundamental research and the development of novel therapeutic strategies.

**Figure 12:**
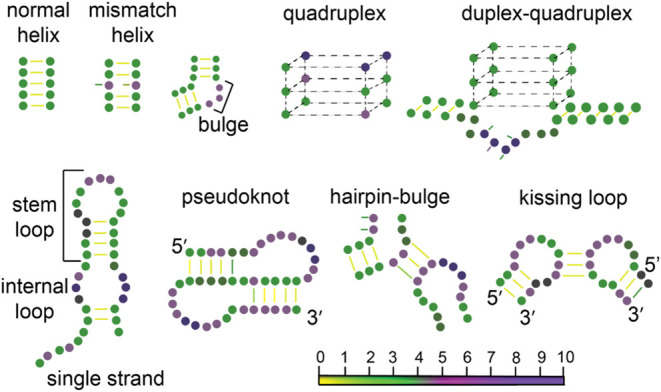
Schematic of DNA (top row) and RNA (bottom row) structural motifs with nucleotides represented as color-coded circles based on PARCH values (scale at the bottom). DNA includes helices, mismatches, and unpaired bases, while RNA features hairpins, bulges, and noncanonical pairings. This schematic illustrates how DNA and RNA structures influence the hydropathy and PARCH values of nucleotides, providing a comparative view of their physicochemical properties across different configurations.

In the case of RNA, the presence of the 2′-hydroxyl group on the ribose sugar further enhances its hydrophilic character compared to DNA. Specifically, PARCH values for the RNA backbone can be as high as 20, while DNA backbones typically have values ranging from 14 to 15. Like DNA, we examined the hydropathy of the structurally diverse RNA motifs such as kissing hairpins, pseudoknots, A-minor motif, tetraloop-tetraloop receptor, triple helices, coaxial stacking, and bulges (Figure 12), influencing overall stability, localization, and function. Some of these features provide unique local hydrophilic character to rRNA and tRNAs.

One of the most striking examples of RNA structural diversity is that seen in ribozymes. The self-splicing ribozyme (Figure 9), the largest molecule in our data set with 388 residues, has three-dimensional regions with high PARCH values in the 15–17 range. These regions allow the ribozyme to position substrates precisely and stabilize reaction intermediates, facilitating essential biochemical reactions.

Moreover, we have shown that riboswitches have pseudoknots and hairpin-loop regions, where, due to the presence of unpaired base pairs, the PARCH values of the RNA backbone are higher. These highly hydrophilic motifs account for the strong interactions of RNA with other charged molecules, metal ions, and proteins, forming ribonucleoprotein complexes.

## Conclusions

In this study, we extended the PARCH scale, originally developed for proteins, to nucleic acids. To accurately capture the distinct chemical properties of the nucleotides, each nucleotide was assigned two PARCH values: one for the sugar–phosphate backbone and another for the nitrogenous base. This dual value accounts for the significant differences between the backbone and the bases, particularly in their interactions with water, and ensures that the unique hydropathy characteristics of each component are preserved. Our analysis revealed several key findings. First, the sugar–phosphate backbone is nearly 1 order of magnitude more hydrophilic than the nitrogenous bases. Second, structural distortions that disrupt base pairing and stacking lead to an increase in local hydrophilicity in DNA. Third, RNA, owing to its structural flexibility and the additional hydroxyl group in its ribose sugar, is more hydrophilic overall than DNA. Fourth, in protein–nucleic acid complexes, proteins are generally less hydrophilic than the nucleotide backbones but exhibit hydropathy levels comparable to those of the nitrogenous bases. Fifth, amino acid residues that interact with nucleic acids in these complexes reduce the hydropathy of the nucleotides in the contact regions. Sixth, charged amino acids, such as lysine and arginine, interact with the phosphate backbone, further lowering hydropathy in the contact zones. Lastly, larger protein structural elements, such as helices, interact extensively with DNA and RNA grooves, engaging directly with the bases rather than the backbones. These findings underscore the importance of adapting the PARCH scale to nucleotides and provide a robust framework for analyzing the hydropathy characteristics of nucleic acids and their interactions. The PARCH scale tool will be applied in future studies to investigate more complex systems, such as nucleosomes, and RNA–protein assemblies, such as spliceosomes and ribosomes, offering valuable insights into their structural and functional properties.

## Supplementary Material



## Data Availability

All structural and computed PARCH values for the protein data set is available at https://github.com/NangiaLab/ParchValuesNucleicAcids/. There is no restriction on the use of the data.

## Notes

List of DNA, RNA, DNA–protein, and RNA–protein structures, simulation parameters for PARCH calculations, radial distribution functions of the RNA statistical analysis of DNA and RNA nucleotides, PARCH data for tRNA, PARCH for rRNA, PARCH data for DNA–protein complex, and PARCH data for RNA–protein complex, statistical analysis of DNA using CHARMM36, OL15, and BSC1 force fields, and PARCH value comparisons of BB region versus phosphate and sugar regions for DNA and RNA (PDF)
