# Supplementary material for: Introducing the PARCH Scale for Quantifying the Hydropathy of Nucleic Acids and Nucleic Acid–Protein Complexes
Source: Langmuir. 2025 Jun 18;41(26):16874–86. doi: 10.1021/acs.langmuir.5c01051 (PMC12257586; doi:10.1021/acs.langmuir.5c01051)
Supplement: Supplementary file 1 [file la5c01051_si_001.pdf]

Supporting Information

Introducing the PARCH Scale for quantifying the hydropathy of nucleic acids and nucleic acid-protein complexes

Ratnakshi Mandal, Jingjing Ji, Claire Nicole Sheridan, Anna M Baur, Andre Christophe Noel, and Shikha Nangia*

Department of Biomedical and Chemical Engineering, Syracuse University, Syracuse, NY 13244, USA

Contents

Table S1: List and short description of DNA structures. 3

Table S2: List and short description of RNA structures. 4

Table S3: Information on DNA-protein and RNA-protein complexes. 5

Table S4: Parameters used for PARCH calculations of nucleic acids 6

Figure S1: Radial distribution functions (RDF) for DNA and Protein. 7

Figure S2: Statistical analysis of DNA nucleotides 8

Figure S3: Statistical analysis of RNA nucleotides 9

Figure S4: Transfer RNA (tRNA) molecule (PDB ID: 7KJU)[38] shown in PARCH colors. 10

Figure S5: Ribosomal RNA (rRNA) molecule (PDB ID: 1K5I)[29] shown in PARCH colors 11

Figure S6: PARCH representation of protein in DNA-protein complex (PDB ID: 4AWL)[52] 12

Figure S7: PARCH representation of protein in RNA-protein complex (PDB ID: 5TF6)[54] 13

Figure S8. Statistical analysis of DNA nucleotides using CHARMM36, OL15, and BSC1 forcefields. 14

Table S5: Comparison of PARCH values of the BB region versus the phosphate and sugar regions for DNA (PDB ID: 8DUT) 15

Table S6: Comparison of PARCH values of the BB region versus the phosphate and sugar regions for RNA (PDB ID: 1YRJ) 17

References.. 18

Table S1: List and short description of DNA structures.

PDB ID	Description
1VJ4	A-DNA double helix octamer[1]
1VT8	Hexagonal form of d(GGGCGCCC)[2]
4H29	B-raf dimer DNA quadruplex[3]
6WJK	Self-assembling DNA crystal with rhombohedral symmetry[4]
6X5D	Dickerson-Drew dodecamer[5]
7EAQ	DNA quadruplex composed of i-motif and Z-DNA (https://www.rcsb.org/structure/7EAQ)
7JKJ	3-D DNA crystal lattice containing J8 immobile Holliday junction with R3 symmetry[6]
7KBW	MYC promoter G-quadruplex[7]
7QA9	10bp DNA/DNA duplex[8]
7V6V	Trimolecular G-quadruplexes[9]
7YVX	Left-handed DNA duplex (https://www.rcsb.org/structure/7YVX)
7ZQL	B-Dodecamer at 3100 bars[10]
8CE2	Adduct formed upon reaction of B-DNA double helical dodecamer with dirhodium tetraacetate[11]
8DUT	Class 1 duplex-G-quadruplex-duplex[12]
8EC1	5'-d(CGCGAARRCGCG)-3' and benzimidazole diamidine complex[13]
8F20	A-tract B-DNA dodecamer[13]
8FDP	Dodecamer[13]
8G4G	One 8-mer DNA[14]
8GH5	Implementing logic gates in DNA crystal engineering[15]
103D	Human centromere d(GGA)$_2$ motif[16]
107D	Covalent duocarmycin A-DNA duplex[17]
108D	DNA complex with fluorescent bis-intercalator TOTO[18]
109D	Bis-bezimidazole compound bound to DNA duplex[19]
111D	DNA duplex containing dA (anti).dG (syn) base pairs[20]
112D	dG-dA base pair in DNA[21]
113D	Guanosine-Thymidine mismatches in B-DNA[22]
114D	Inosine-Adenine base pairs in B-DNA duplex[23]
115D	A-DNA octamer[24]
117D	Alternating dodecamer in the A-DNA form[25]
119D	d(CGTAGATCTACG) at 2.25 Å resolution[26]

Table S2: List and short description of RNA structures.

PDB ID	Description
1DUH	Conserved domain of 4.5S RNA[27]
1ELH	Helix I from 5S RNA[28]
1K5I	Ribosomal RNA hairpin[29]
1P6V	tRNA domain of transfer-messenger RNA[30]
1YRJ	Ribosomal rRNA A site[31]
2PCV	rRNA substrate bound to snoRNA[32]
3BO0	Ribosome-secY complex[33]
6BGB	16mer GCAGNCUUAAGUCUGC[34]
6TB7	ADP-binding domain of the NAD+ riboswitch[35]
6UBU	Guanine riboswitch[36]
7DMQ	crRNA-anti tag RNA complex[37]
7K4L	DENV1 SLA bottom stem RNA (https://www.rcsb.org/structure/7K4L)
7KJU	Cgi121-tRNA complex[38]
7Q6L	RNA G-quadruplex (https://www.rcsb.org/structure/7Q6L)
7SXP	G-quadruplex structure in NRAS mRNA[39]
7U4A	*Zika* virus xrRNA1 (https://www.rcsb.org/structure/7U4A)
7UMC	DENV1 SLA RNA[40]
7V06	HIV Splice Site A3[41]
7X34	RNC-RAC complex[42]
7YC8	*Tetrahymena* ribozyme[43]
8BU8	Single-stranded paranemic crossover RNA triangle[44]
8BWT	Stem-loop 1 in the 3'-UTR of the SARS-CoV2 genomic RNA[45]
8CQ1	Stem-loop 4 of the 5'-UTR of the SARS-CoV2 genomic RNA[46]
8D28	Theophylline aptamer[47]
8FCS	Pre-miR-31[48]
8S95	Poliovirus cloverleaf RNA with tRNA scaffold[49]
8SA5	Adenosylcobalamin-bound riboswitch[50]
8SP9	Coxsackievirus B3 cloverleaf RNA with tRNA scaffold[49]
8TNS	Poly(UG) RNA (GU)12 G-quadruplex[51]

Table S3: Information on DNA-protein and RNA-protein complexes.

PDB ID	Description
4AWL	NF-Y transcription factor[52]
1G1X	Ribosomal proteins S15, S6, S18, and 16S ribosomal RNA[53]
5TF6	U6 small nuclear ribonucleoprotein core[54]

Table S4: Parameters used for PARCH calculations of nucleic acids

Parameter	Description	Protein	Nucleic Acids
d_{max}	Distance between the center of geometry of the molecule to the farthest point on the protein surface	Depends on size of molecule	Depends on size of molecule
$d_{interion}$	Maximum distance between any two counterions	3.0 nm	3.0 Å
d_{ion}	Radial distance between surface of molecule and counterions	3.0 nm	Minimum 3.0 Å, smallest distance that accommodates all counter-ions with $d_{interion}$.
d_b	Distance between counterion to box boundary	3.0 nm	3.0 Å
l	Length of simulation box	$2\times(d_{max}+d_{ion}+d_b)$	$2\times(d_{max}+d_{ion}+d_b)$
d_{shell}	Water layer thickness from the surface of the molecule, when solvated	4.15 Å	4.5 Å
d_{water}	Cut-off used to calculate the number of water molecules around each residue	3.15 Å	4.5 Å

Figure S1: Radial distribution functions (RDF) for DNA and Protein.

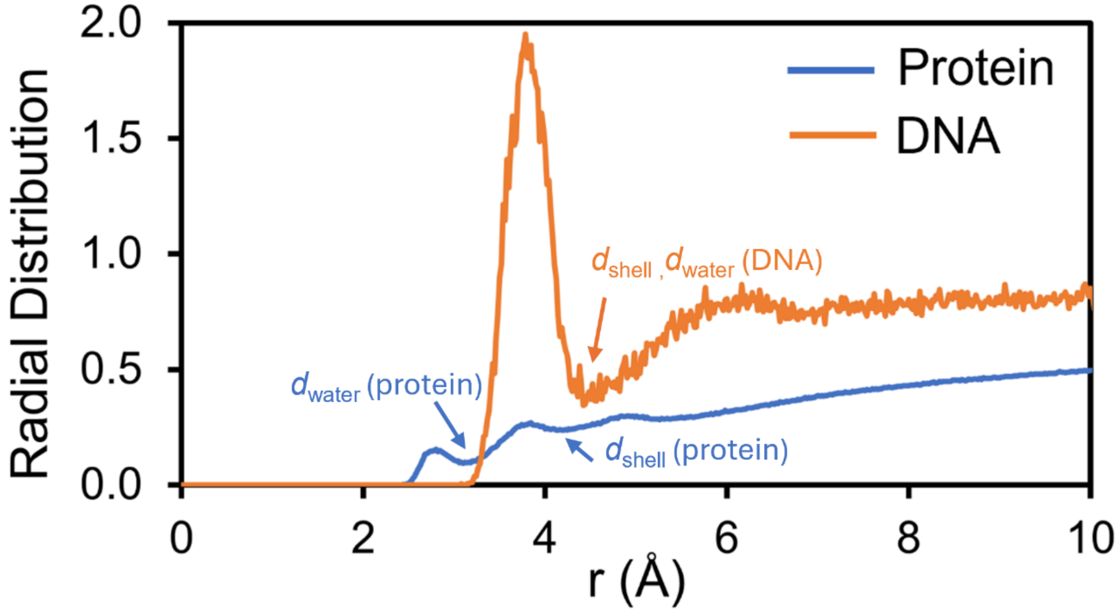

The figure illustrates the radial distribution function (RDF) of the backbone Cα of the protein (blue) and the phosphate (P) in the DNA backbone (orange), with oxygen (O) from water as the reference. The values for d_{shell} and d_{water} for PARCH calculations were derived from the RDF analysis of the protein and DNA. For the protein, d_{shell} was set to 4.15 Å, corresponding to the second radial node of the RDF curve, while d_{water} was set to 3.15 Å, based on the first radial node. For DNA, the RDF had a single node at 4.5 Å, so both d_{shell} and d_{water} was set at 4.5 Å.

Figure S2: Statistical analysis of DNA nucleotides

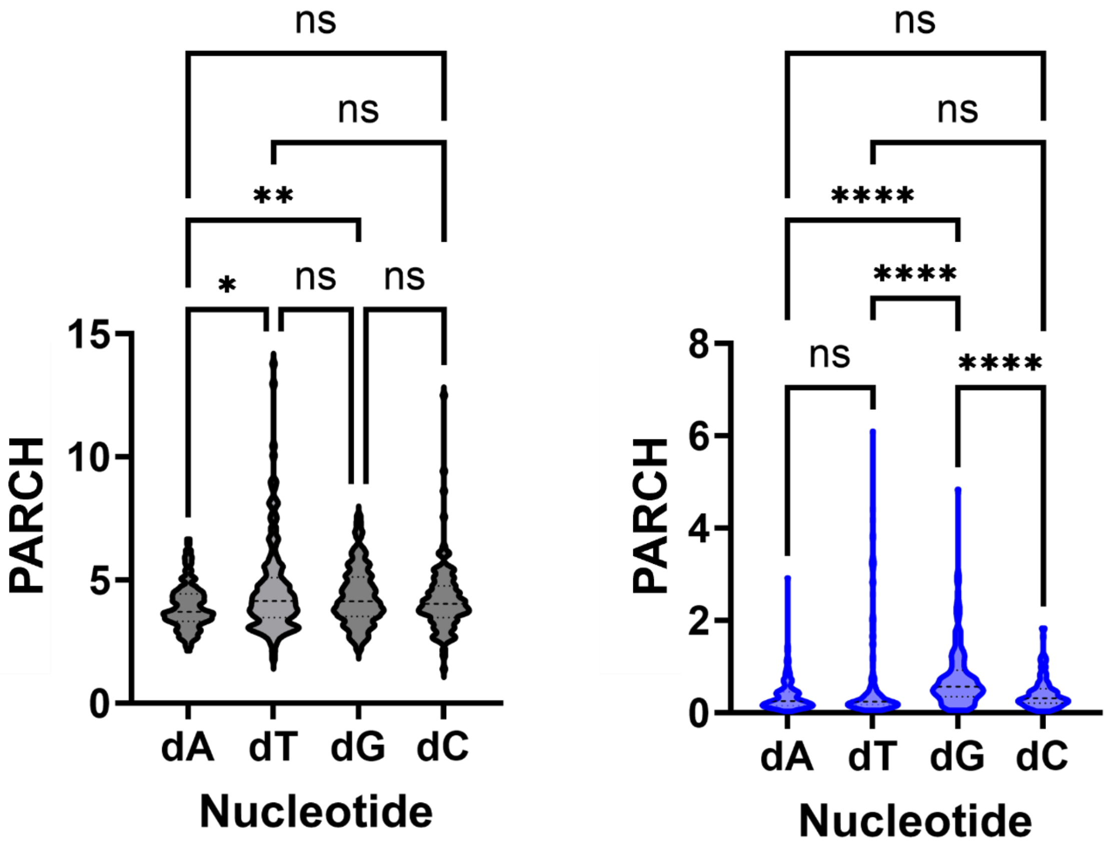

Violin plots comparing the distribution of PARCH values for DNA backbone (left, gray) and base (right, blue) regions across nucleotide types. For the backbone, the PARCH values are tightly distributed with higher central values, reflecting the hydrophilic nature of the DNA backbone. In contrast, the base PARCH values show a wider range, with lower central values indicating their hydrophobic character. Notably, the backbone PARCH value distribution for dA differs significantly from those of dT and dG, while the base PARCH value distribution for dG stands out significantly compared to the other bases. These differences highlight variations in the hydropathy profiles among nucleotides

To assess whether the observed differences in dG abundance across groups are statistically significant, we applied the Kruskal–Wallis test—a non-parametric method that evaluates whether the distributions of ranked values differ among multiple groups. The test does not assume normality and is sensitive to systematic shifts in rank distributions. In this case, the consistently higher abundance of dG in certain groups, particularly in the NB group, results in rank shifts that are sufficient to yield statistical significance. This is visually supported by the violin plots, where dG shows a broader and elevated distribution compared to other nucleotides.

Figure S3: Statistical analysis of RNA nucleotides

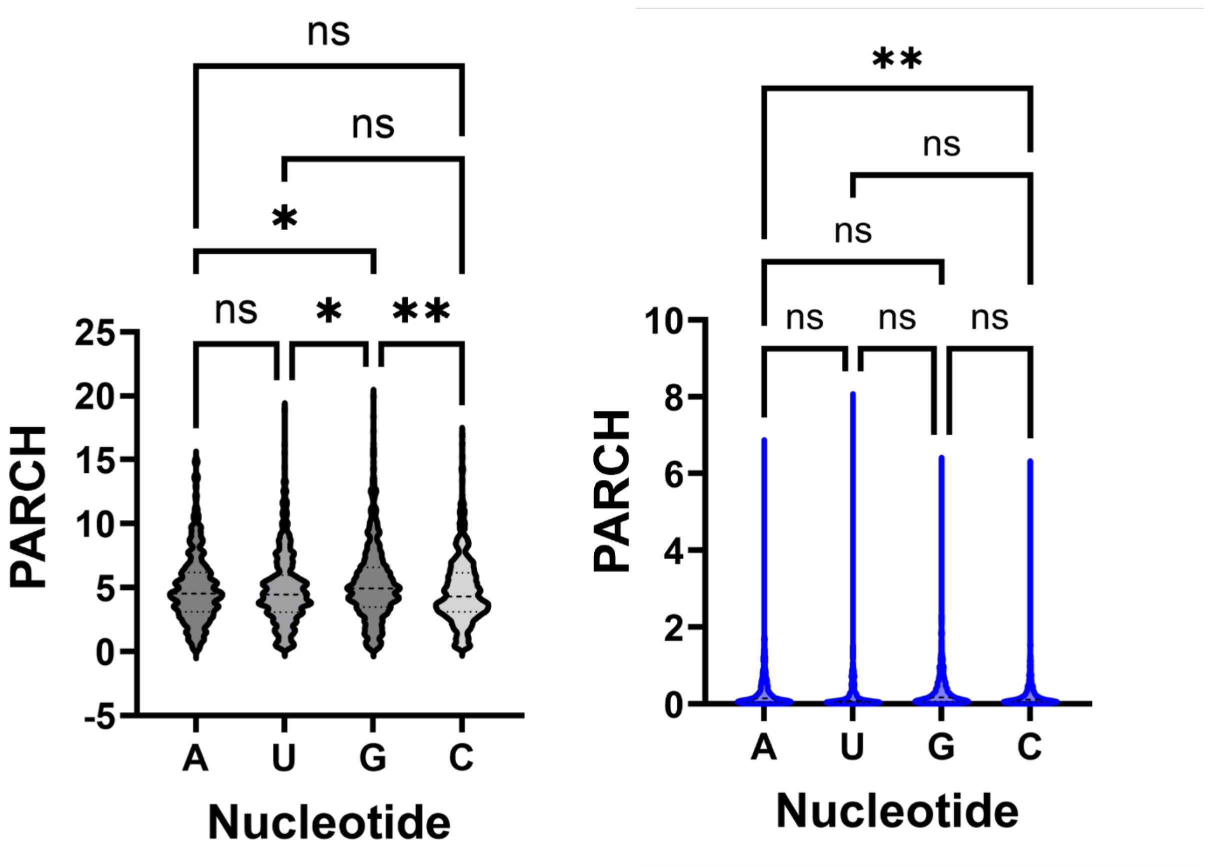

Violin plot distributions of PARCH values of RNA nucleotide backbone (left, gray) and bases (right, blue). Each violin plot represents a specific nucleotide type (A, U, G, C). Significance testing indicates notable differences between nucleotides. In the backbone, G exhibits significantly higher PARCH values compared to A (*), U (*), and C (**), reflecting variability in backbone hydropathy among nucleotides, while other pairwise comparisons are non-significant. In the bases, the PARCH values for A and C are significantly higher (**), while other nucleotides show no significant differences (ns).

Similar to the analysis performed for dG in the DNA dataset, we evaluated whether there were significant differences in guanine (G) nucleotide abundance in the RNA data. Using the Kruskal–Wallis test, we found that G consistently exhibits higher abundance, leading to a statistically significant difference—particularly evident in the PARCH value distribution within the NB region for the G nucleotide.

Figure S4: Transfer RNA (tRNA) molecule (PDB ID: 7KJU)[38] shown in PARCH colors.

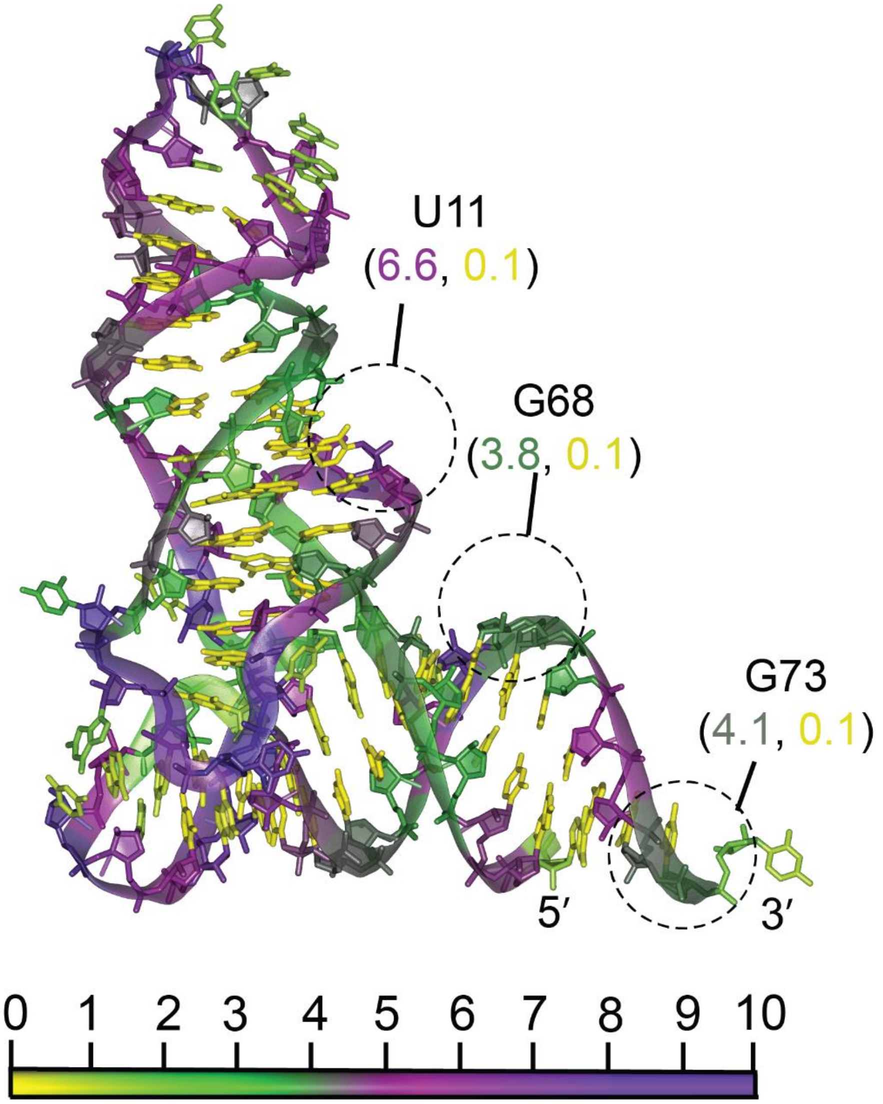

The figure highlights key residues like U11, G68 and G73 which have been experimentally determined to bind to protein residues. Compared to mRNA and rRNA, the structure of tRNA is highly complex, which leads to more regions of high PARCH values.

Figure S5: Ribosomal RNA (rRNA) molecule (PDB ID: 1K5I)[29] shown in PARCH colors

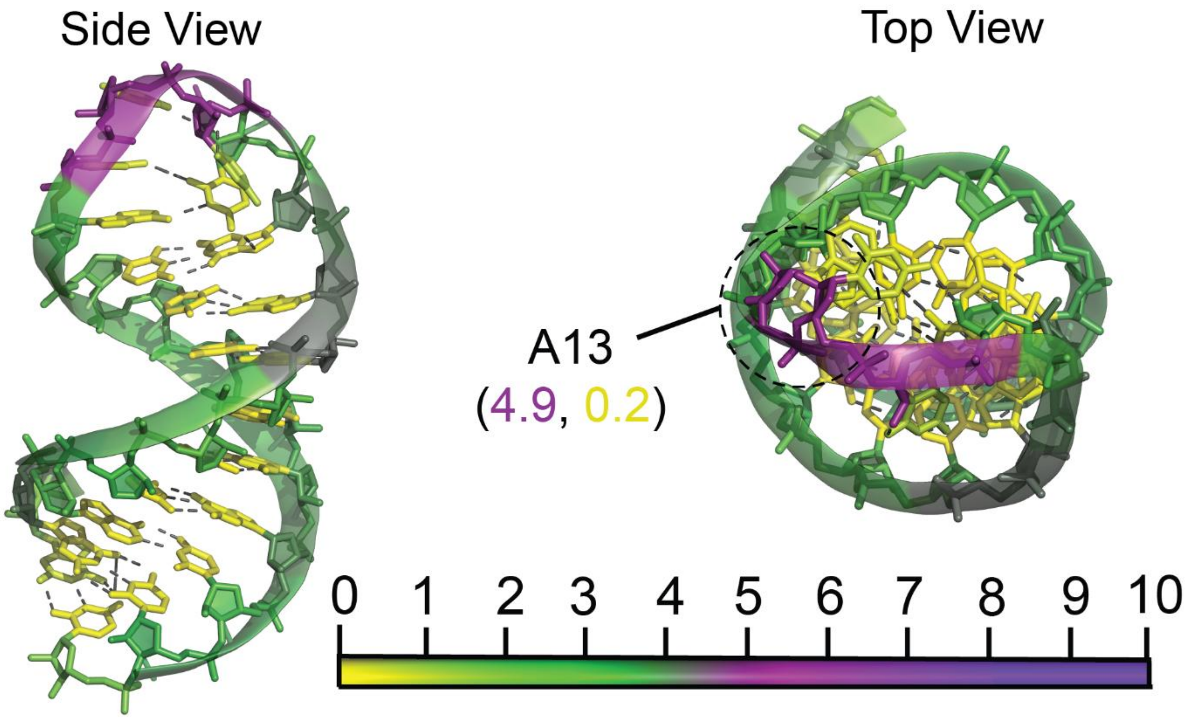

The rRNA has a hairpin secondary structure, and a key residue in this hairpin is highlighted. The residue A13 has a mismatched base pairing, which results in high PARCH values, compared to the rest of the molecule, as shown in the figure.

Figure S6: PARCH representation of protein in DNA-protein complex (PDB ID: 4AWL)[52]

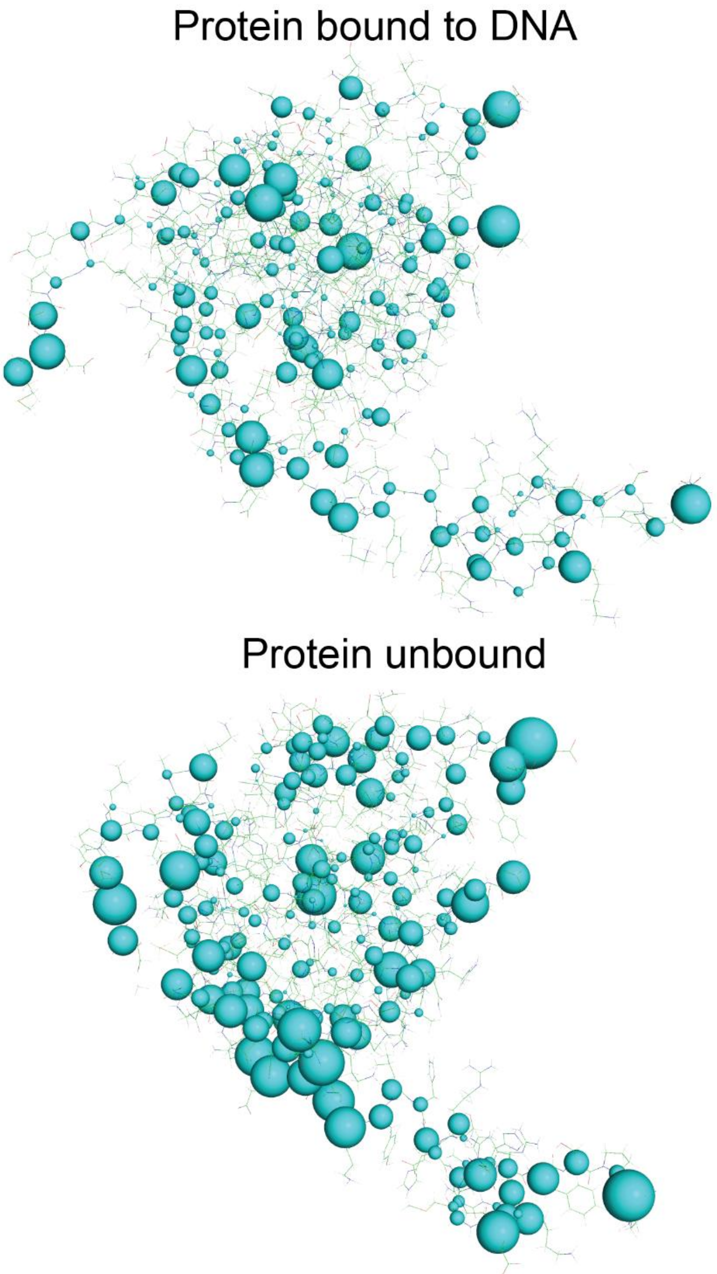

The figure presents the PARCH values of amino acids in a protein, represented as bubbles, under two conditions: when the protein is bound to DNA (top) and when it is unbound (bottom). The radius of each bubble is directly proportional to the PARCH value (PV/5). For clarity, the DNA is omitted from the panels. The C_α of the amino acid is shown in bubbles and the other atoms are shown as sticks. The figure demonstrates that the PARCH values of amino acids are reduced when the protein is bound to DNA.

Figure S7: PARCH representation of protein in RNA-protein complex (PDB ID: 5TF6)[54]

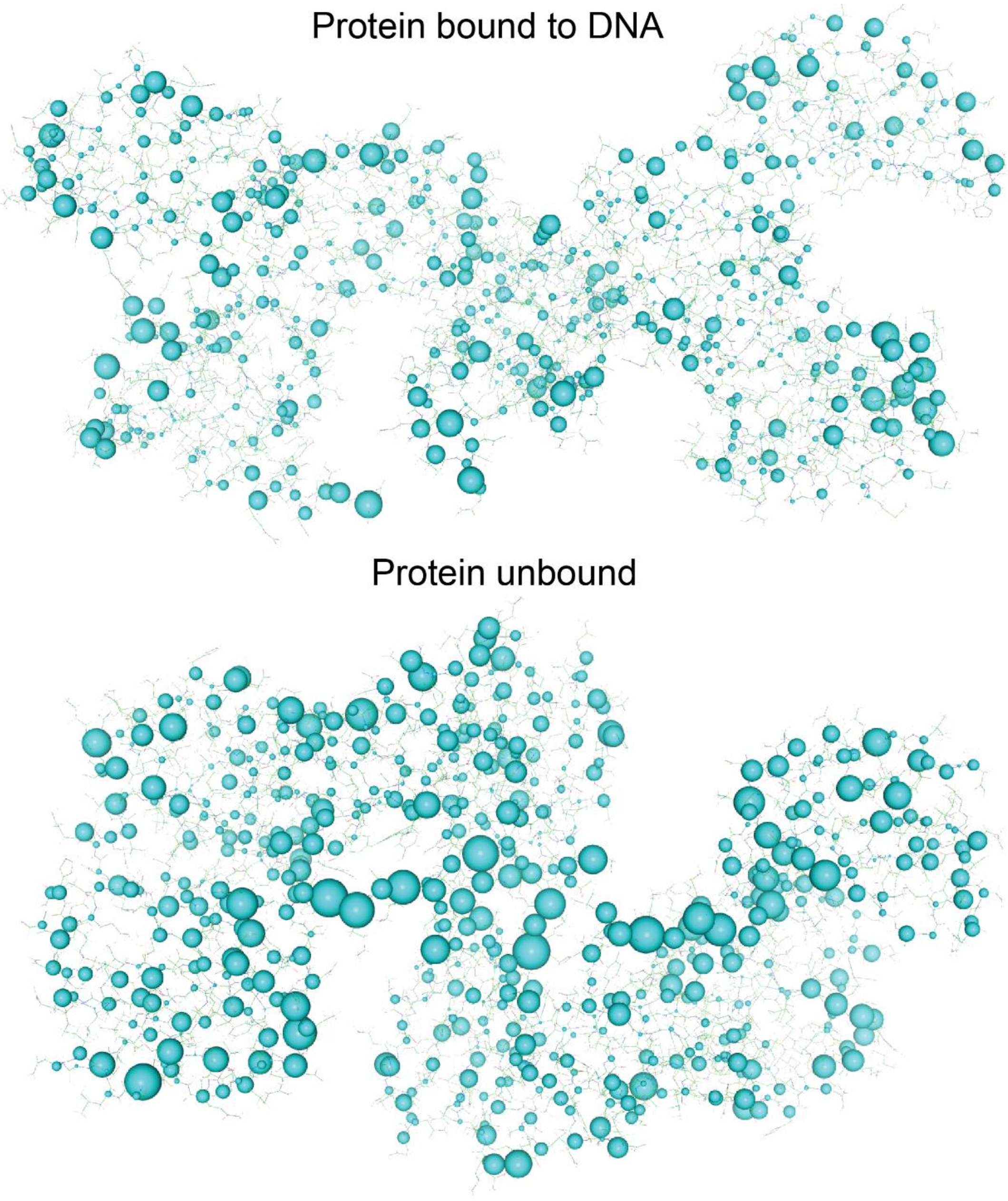

The figure presents the PARCH values of amino acids in a protein, represented as bubbles (cyan), under two conditions: when the protein is bound to RNA (top) and when it is unbound (bottom). The radius of each bubble is directly proportional to the PARCH value (PV/5). For clarity, the RNA is omitted from the panels. The C_α of the amino acid is shown in bubbles and the other atoms are shown as sticks. The figure demonstrates that the PARCH values of amino acids are reduced when the protein is bound to RNA.

Figure S8. Statistical analysis of DNA nucleotides using CHARMM36, OL15, and BSC1 forcefields.
Violin plot distributions of PARCH values of DNA nucleotide backbone (gray) and bases (blue).

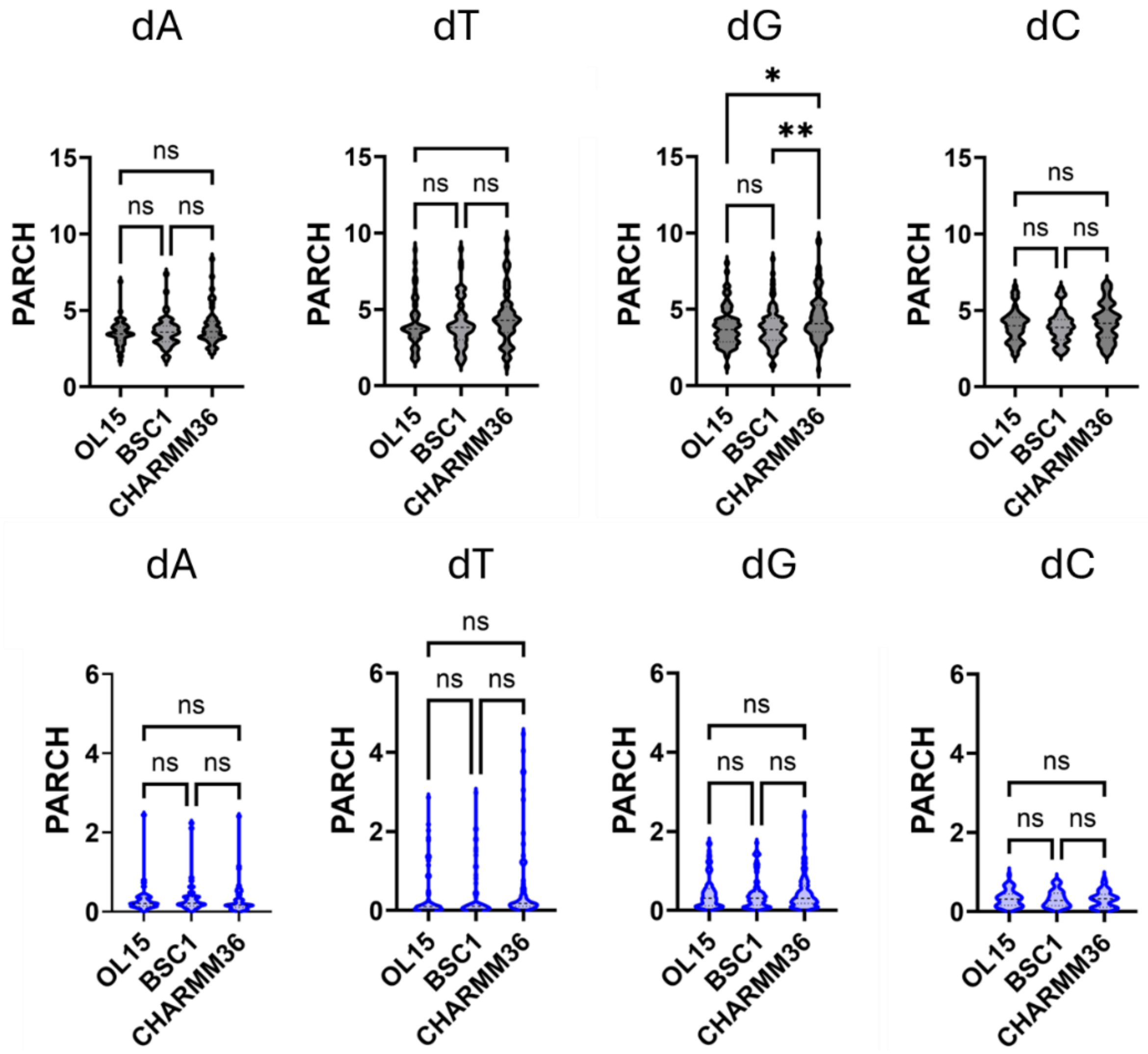

Table S5: Comparison of PARCH values of the BB region versus the phosphate and sugar regions for DNA (PDB ID: 8DUT)

The molecules has been equilibrated for 10 ns before annealing.

ChainID	Resname	PHO	SUG	PHO+SUG	BB	Δ=BB–(PHO+SUG)
A1	DG	0.0	3.9	3.9	4.8	0.9
A2	DC	1.0	1.7	2.8	4.2	1.4
A3	DG	0.9	1.8	2.7	5.1	2.4
A4	DT	0.7	1.5	2.3	4.4	2.1
A5	DG	0.6	1.3	1.9	3.8	1.9
A6	DC	0.8	1.6	2.4	4.2	1.8
A7	DC	1.1	1.1	2.1	3.9	1.7
A8	DG	0.5	0.5	1.0	2.2	1.3
A9	DC	0.3	0.6	0.8	1.9	1.1
A10	DA	0.6	0.5	1.1	2.1	1.0
A11	DT	0.3	1.1	1.5	3.2	1.8
A12	DT	1.4	1.4	2.8	5.6	2.8
A13	DA	1.6	0.9	2.5	3.6	1.1
A14	DA	1.0	0.7	1.8	3.4	1.7
A15	DT	0.7	0.7	1.4	2.9	1.5
A16	DT	0.6	1.2	1.8	3.5	1.7
A17	DT	1.1	0.9	2.0	3.5	1.5
A18	DT	2.0	3.8	5.9	8.3	2.4
A19	DT	1.9	3.2	5.1	9.0	3.9
A20	DT	1.7	0.8	2.5	3.6	1.1
A21	DT	0.5	4.6	5.1	6.6	1.5
A22	DT	2.5	6.2	8.7	13.0	4.3
A23	DT	1.1	4.7	5.8	10.4	4.6
A24	DT	2.2	2.3	4.5	8.7	4.2
A25	DT	1.7	3.9	5.6	8.1	2.5
A26	DT	1.2	2.5	3.7	7.1	3.4
A27	DT	1.5	3.9	5.4	9.0	3.6
A28	DT	1.9	9.2	11.1	13.8	2.7
A29	DT	2.2	3.2	5.4	8.0	2.6
A30	DT	2.1	3.8	5.9	10.0	4.1
A31	DT	2.0	2.9	4.9	7.5	2.6
A32	DT	1.3	1.0	2.3	3.8	1.6
A33	DT	0.6	0.6	1.2	2.7	1.4
A34	DT	0.4	0.7	1.1	2.4	1.3
A35	DT	0.6	0.8	1.4	3.5	2.1
A36	DT	0.9	1.3	2.2	4.9	2.7
A37	DG	1.1	1.4	2.4	5.2	2.7
A38	DT	1.0	1.3	2.3	4.1	1.9
A39	DA	0.8	0.9	1.7	3.8	2.1
A40	DT	0.9	0.8	1.7	3.7	1.9
A41	DA	0.9	1.1	2.0	4.4	2.4
A42	DC	0.9	1.1	2.0	4.2	2.2
A43	DA	1.2	2.0	3.1	5.0	1.9
A44	DT	1.2	2.1	3.3	5.8	2.5
A45	DA	1.5	2.0	3.5	6.5	3.0

ChainID	Resname	PHO	SUG	PHO+SUG	BB	BB - (PHO+SUG)
A46	DG	2.0	2.3	4.2	6.2	1.9
B47	DC	0.0	0.7	0.7	1.3	0.6
B48	DT	1.2	2.1	3.3	4.5	1.2
B49	DA	0.8	1.7	2.5	5.6	3.2
B50	DT	1.1	1.6	2.7	4.8	2.1
B51	DG	0.5	1.7	2.2	4.9	2.7
B52	DT	1.4	1.5	2.8	5.1	2.2
B53	DA	0.7	1.0	1.7	4.4	2.7
B54	DT	1.1	1.0	2.1	4.0	1.8
B55	DA	0.5	0.9	1.4	3.2	1.8
B56	DC	0.9	1.0	1.9	3.5	1.6
B57	DA	0.3	0.7	0.9	2.5	1.6
B58	DA	0.6	1.8	2.4	3.9	1.5
B59	DA	0.5	1.7	2.2	5.1	2.9
B60	DG	1.3	1.7	3.0	4.8	1.8
B61	DA	1.1	0.9	2.0	4.1	2.1
B62	DG	1.2	0.9	2.1	4.5	2.5
B63	DG	0.7	1.4	2.1	4.8	2.7
B64	DG	1.0	1.4	2.4	4.5	2.1
B65	DT	1.1	3.0	4.1	7.1	2.9
B66	DG	1.0	1.0	2.1	5.2	3.2
B67	DG	1.5	2.1	3.6	5.6	2.0
B68	DG	1.1	2.1	3.2	5.8	2.6
B69	DT	0.6	5.0	5.6	8.1	2.5
B70	DA	1.1	3.3	4.4	6.2	1.7
B71	DG	0.6	1.7	2.3	3.4	1.2
B72	DG	1.2	2.0	3.3	5.9	2.6
B73	DG	1.2	2.3	3.5	6.3	2.8
B74	DT	1.1	2.3	3.4	6.6	3.2
B75	DG	0.2	0.8	1.0	2.4	1.4
B76	DG	2.3	2.6	4.9	7.2	2.4
B77	DG	0.6	1.0	1.6	3.4	1.8
B78	DT	0.4	0.6	1.0	1.8	0.8
B79	DT	0.5	0.6	1.1	2.1	1.0
B80	DT	0.7	1.0	1.7	2.9	1.2
B81	DA	0.6	1.5	2.1	4.4	2.3
B82	DA	0.9	1.7	2.5	5.0	2.4
B83	DT	1.0	1.3	2.2	4.4	2.2
B84	DG	0.7	1.0	1.7	3.7	2.0
B85	DC	1.1	1.1	2.2	4.0	1.8
B86	DG	0.9	0.8	1.7	3.2	1.6
B87	DG	1.0	0.7	1.6	3.1	1.5
B88	DC	0.6	0.8	1.4	3.1	1.7
B89	DA	0.9	0.9	1.8	3.5	1.7
B90	DC	0.9	1.1	2.1	3.8	1.8
B91	DG	0.6	1.1	1.6	4.0	2.4
B92	DC	0.8	2.0	2.8	3.9	1.1

Table S6: Comparison of PARCH values of the BB region versus the phosphate and sugar regions for RNA (PDB ID: 1YRJ)

The molecule has been equilibrated for 10 ns before annealing.

ChainID	Resname	PHO	SUG	PHO+SUG	BB	Δ=BB–(PHO+SUG)
A1	U	4.7	1.3	5.9	6.0	0.0
A2	U	3.0	2.4	5.5	6.8	1.4
A3	G	2.0	3.2	5.2	7.3	2.1
A4	C	2.3	3.2	5.5	6.6	1.1
A5	G	1.2	2.7	3.9	5.2	1.3
A6	U	1.2	1.8	3.0	4.0	1.0
A7	C	1.1	2.0	3.1	4.1	1.1
A8	A	1.0	1.7	2.7	3.5	0.9
A9	C	0.9	2.0	2.9	3.8	0.9
A10	A	1.1	2.6	3.7	4.5	0.8
A11	C	1.0	3.1	4.0	4.5	0.4
A12	C	1.1	2.8	3.9	4.5	0.6
A13	G	0.9	2.8	3.7	4.5	0.8
A14	G	1.1	2.7	3.8	4.5	0.7
A15	U	1.3	2.8	4.1	4.8	0.7
A16	G	1.7	3.1	4.7	6.0	1.3
A17	A	2.6	3.8	6.5	7.9	1.5
A18	A	2.2	2.9	5.1	5.5	0.4
A19	G	1.2	3.1	4.3	5.4	1.1
A20	U	1.1	3.4	4.5	5.3	0.8
A21	C	1.2	3.5	4.7	5.8	1.1
A22	G	1.3	3.1	4.4	5.1	0.7
A23	C	1.0	1.7	2.6	3.6	1.0
B25	U	3.2	0.7	3.9	4.0	0.1
B26	G	2.2	2.7	4.9	6.4	1.5
B27	C	2.3	2.8	5.0	6.1	1.0
B28	G	2.0	3.2	5.2	6.4	1.2
B29	U	1.0	2.3	3.3	4.8	1.5
B30	C	0.7	1.6	2.3	3.5	1.2
B31	A	1.0	1.4	2.4	3.6	1.2
B32	C	1.2	2.0	3.3	4.5	1.2
B33	A	1.2	2.6	3.8	4.8	1.1
B34	C	1.2	2.6	3.9	4.8	1.0
B35	C	1.3	3.0	4.3	5.1	0.8
B36	G	1.3	2.9	4.2	4.8	0.6
B37	G	1.0	2.9	3.9	5.2	1.3
B38	U	1.1	3.1	4.1	5.1	0.9
B39	G	1.5	2.8	4.3	5.3	1.0
B40	A	1.9	2.5	4.4	6.2	1.8
B41	A	2.5	3.4	5.8	7.1	1.3
B42	G	1.5	3.3	4.9	5.5	0.6
B43	U	1.2	3.4	4.7	5.6	1.0
B44	C	1.7	3.9	5.6	7.2	1.6
B45	G	1.5	2.9	4.4	5.6	1.2
B46	C	0.9	1.4	2.3	3.1	0.8

References

(1) Shakked, Z.; Rabinovich, D.; Kennard, O.; Cruse, W. B. T.; Salisbury, S. A.; Viswamitra, M. A. Sequence-dependent conformation of an A-DNA double helix: The crystal structure of the octamer d(G-G-T-A-T-A-C-C). *Journal of Molecular Biology* **1983**, *166* (2), 183-201. DOI: https://doi.org/10.1016/S0022-2836(83)80005-9.

(2) Shakked, Z.; Guerstein-Guzikevich, G.; Eisenstein, M.; Frolow, F.; Rabinovich, D. The conformation of the DNA double helix in the crystal is dependent on its environment. *Nature* **1989**, *342* (6248), 456-460. DOI: 10.1038/342456a0.

(3) Wei, D.; Todd, A. K.; Zloh, M.; Gunaratnam, M.; Parkinson, G. N.; Neidle, S. Crystal structure of a promoter sequence in the B-*raf* gene reveals an intertwined dimer quadruplex. *Journal of the American Chemical Society* **2013**, *135* (51), 19319-19329. DOI: 10.1021/ja4101358.

(4) Simmons, C. R.; MacCulloch, T.; Zhang, F.; Liu, Y.; Stephanopoulos, N.; Yan, H. A self-assembled rhombohedral DNA crystal scaffold with tunable cavity sizes and high-resolution structural detail. *Angewandte Chemie International Edition* **2020**, *59* (42), 18619-18626. DOI: https://doi.org/10.1002/anie.202005505.

(5) Chen, C.; Fang, Z.; Huang, Z. 2′-β-selenium atom on thymidine to control β-form DNA conformation and large crystal formation. *Crystal Growth & Design* **2022**, *22* (6), 3601-3604. DOI: 10.1021/acs.cgd.2c00474.

(6) Simmons, C. R.; MacCulloch, T.; Krepl, M.; Matthies, M.; Buchberger, A.; Crawford, I.; Šponer, J.; Šulc, P.; Stephanopoulos, N.; Yan, H. The influence of Holliday junction sequence and dynamics on DNA crystal self-assembly. *Nature Communications* **2022**, *13* (1), 3112. DOI: 10.1038/s41467-022-30779-6.

(7) Dickerhoff, J.; Dai, J.; Yang, D. Structural recognition of the *MYC* promoter G-quadruplex by a quinoline derivative: insights into molecular targeting of parallel G-quadruplexes. *Nucleic Acids Research* **2021**, *49* (10), 5905-5915. DOI: 10.1093/nar/gkab330

(8) Li, Q.; Trajkovski, M.; Fan, C.; Chen, J.; Zhou, Y.; Lu, K.; Li, H.; Su, X.; Xi, Z.; Plavec, J.; et al. 4′-SCF3-labeling constitutes a censitive 19F NMR probe for characterization of cnteractions in the inor groove of DNA. *Angewandte Chemie International Edition* **2022**, *61* (47), e202201848. DOI: https://doi.org/10.1002/anie.202201848.

(9) Hu, W.; Jing, H.; Fu, W.; Wang, Z.; Zhou, J.; Zhang, N. Conversion to trimolecular G-quadruplex by spontaneous Hoogsteen pairing-based strand displacement reaction between bimolecular G-quadruplex and double G-rich probes. *Journal of the American Chemical Society* **2023**, *145* (33), 18578-18590. DOI: 10.1021/jacs.3c05617.

(10) Prangé, T.; Colloc'h, N.; Dhaussy, A.-C.; Lecouvey, M.; Migianu-Griffoni, E.; Girard, E. Behavior of B- and Z-DNA crystals under high hydrostatic pressure. In *Crystals*, 2022; Vol. 12.

(11) Tito, G.; Troisi, R.; Ferraro, G.; Geri, A.; Massai, L.; Messori, L.; Sica, F.; Merlino, A. Dirhodium tetraacetate binding to a B-DNA double helical dodecamer probed by X-ray crystallography and mass spectrometry. *Dalton Transactions* **2023**, *52* (21), 6992-6996, 10.1039/D3DT00320E. DOI: 10.1039/D3DT00320E.

(12) Monsen, R. C.; Chua, Eugene Y. D.; Hopkins, Jesse B.; Chaires, Jonathan B.; Trent, John O. Structure of a 28.5 kDa duplex-embedded G-quadruplex system resolved to 7.4 Å resolution with cryo-EM. *Nucleic Acids Research* **2023**, *51* (4), 1943-1959. DOI: 10.1093/nar/gkad014 (acccessed 12/6/2024).

(13) Ogbonna, E. N.; Paul, A.; Farahat, A. A.; Terrell, J. R.; Mineva, E.; Ogbonna, V.; Boykin, D. W.; Wilson, W. D. X-ray structure characterization of the selective recognition of at base pair sequences. *ACS Bio & Med Chem Au* **2023**, *3* (4), 335-348. DOI: 10.1021/acsbiomedchemau.3c00002.

(14) Zhao, J.; Zhang, C.; Lu, B.; Sha, R.; Noinaj, N.; Mao, C. Divergence and convergence: Complexity emerges in crystal engineering from an 8-mer DNA. *Journal of the American Chemical Society* **2023**, *145* (19), 10475-10479. DOI: 10.1021/jacs.3c01941.

(15) Zhang, C.; Paluzzi, V. E.; Sha, R.; Jonoska, N.; Mao, C. Implementing logic gates by DNA crystal engineering. *Advanced Materials* **2023**, *35* (33), 2302345. DOI: https://doi.org/10.1002/adma.202302345 (acccessed 2024/12/06).

(16) Chou, S.-H.; Zhu, L.; Reid, B. R. The unusual structure of the human centromere (gga)2 motif: Unpaired guanosine residues stacked between sheared g·a pairs. *Journal of Molecular Biology* **1994**, *244* (3), 259-268. DOI: https://doi.org/10.1006/jmbi.1994.1727.

(17) Lin, C. H.; Patel, D. J. Solution structure of the covalent duocarmycin A-DNA duplex complex. *Journal of Molecular Biology* **1995**, *248* (1), 162-179. DOI: https://doi.org/10.1006/jmbi.1995.0209.

(18) Spielmann, H. P.; Wemmer, D. E.; Jacobsen, J. P. Solution structure of a DNA complex with the fluorescent bis-intercalator TOTO determined by NMR spectroscopy. *Biochemistry* **1995**, *34* (27), 8542-8553. DOI: 10.1021/bi00027a004.

(19) Wood, A. A.; Nunn, C. M.; Czarny, A.; Boykin, D. W.; Neidle, S. Variability in DNA minor groove width recognised by ligand binding: the crystal structure of a bis-benzimidazole compound bound to the DNA duplex d(CGCGAATTCGCG)2. *Nucleic Acids Research* **1995**, *23* (18), 3678-3684. DOI: 10.1093/nar/23.18.3678.

(20) Brown, T.; Leonard, G. A.; Booth, E. D.; Chambers, J. Crystal structure and stability of a DNA duplex containing A(anti) · G(syn) base-pairs. *Journal of Molecular Biology* **1989**, *207* (2), 455-457. DOI: https://doi.org/10.1016/0022-2836(89)90268-4.

(21) Brown, T.; Hunter, W. N.; Kneale, G.; Kennard, O. Molecular structure of the G.A base pair in DNA and its implications for the mechanism of transversion mutations. *Proceedings of the National Academy of Sciences* **1986**, *83* (8), 2402-2406. DOI: 10.1073/pnas.83.8.2402.

(22) Hunter, W. N.; Brown, T.; Kneale, G.; Anand, N. N.; Rabinovich, D.; Kennard, O. The structure of guanosine-thymidine mismatches in B-DNA at 2.5-A resolution. *The Journal of biological chemistry* **1987**, *262* (21), 9962-9970. DOI: 10.2210/pdb113d/pdb From NLM.

(23) Corfield, p. W. R.; Hunter, W. N.; Brown, T.; Robinson, P.; Kennard, O. Inosine.adenine base pairs in a B-DNA duplex. *Nucleic Acids Research* **1987**, *15* (19), 7935-7949. DOI: 10.1093/nar/15.19.7935.

(24) Kennard, O.; Cruse, W. B. T.; Nachman, J.; Prange, T.; Shakked, Z.; Rabinovich, D. Ordered water structure in an A-DNA octamer at 1.7Å resolution. *Journal of Biomolecular Structure and Dynamics* **1986**, *3* (4), 623-647. DOI: 10.1080/07391102.1986.10508452.

(25) Bingman, C.; Jain, S.; Zon, G.; Sundaralingam, M. Crystal and molecular structure of the alternating dodecamer d(GCGTACGTACGC) in the A-DNA form: comparison with the isomorphous non-alternating dodecamer d(CCGTACGTACGG). *Nucleic Acids Research* **1992**, *20* (24), 6637-6647. DOI: 10.1093/nar/20.24.6637.

(26) Leonard, G. A.; Hunter, W. N. Crystal and molecular structure of d(CGTAGATCTACG) at 2·25 Å resolution. *Journal of Molecular Biology* **1993**, *234* (1), 198-208. DOI: https://doi.org/10.1006/jmbi.1993.1574.

(27) Jovine, L.; Hainzl, T.; Oubridge, C.; Scott, W. G.; Li, J.; Sixma, T. K.; Wonacott, A.; Skarzynski, T.; Nagai, K. Crystal structure of the Ffh and EF-G binding sites in the conserved domain IV of Escherichia coli 4.5S RNA. *Structure* **2000**, *8* (5), 527-540. DOI: https://doi.org/10.1016/S0969-2126(00)00137-4.

(28) White, S. A.; Nilges, M.; Huang, A.; Brunger, A. T.; Moore, P. B. NMR analysis of helix I from the 5S RNA of Escherichia coli. *Biochemistry* **1992**, *31* (6), 1610-1621. DOI: 10.1021/bi00121a005.

(29) Nagaswamy, U.; Gao, X.; Martinis, S. A.; Fox, G. E. NMR structure of a ribosomal RNA hairpin containing a conserved CUCAA pentaloop. *Nucleic Acids Research* **2001**, *29* (24), 5129-5139. DOI: 10.1093/nar/29.24.5129.

(30) Gutmann, S.; Haebel, P. W.; Metzinger, L.; Sutter, M.; Felden, B.; Ban, N. Crystal structure of the transfer-RNA domain of transfer-messenger RNA in complex with SmpB. *Nature* **2003**, *424* (6949), 699-703. DOI: 10.1038/nature01831.
(31) Han, Q.; Zhao, Q.; Fish, S.; Simonsen, K. B.; Vourloumis, D.; Froelich, J. M.; Wall, D.; Hermann, T. Molecular recognition by glycoside pseudo base pairs and triples in an apramycin–RNA complex. *Angewandte Chemie International Edition* **2005**, *44* (18), 2694-2700. DOI: https://doi.org/10.1002/anie.200500028.
(32) Jin, H.; Loria, J. P.; Moore, P. B. Solution structure of an rRNA substrate bound to the pseudouridylation pocket of a box H/ACA snoRNA. *Molecular Cell* **2007**, *26* (2), 205-215. DOI: https://doi.org/10.1016/j.molcel.2007.03.014.
(33) Makabe, K.; Nakamura, T.; Kuwajima, K. Structural insights into the stability perturbations induced by N-terminal variation in human and goat α-lactalbumin. *Protein Engineering, Design and Selection* **2013**, *26* (2), 165-170. DOI: 10.1093/protein/gzs093 (acccessed 12/6/2024).
(34) Suter, S. R.; Ball-Jones, A.; Mumbleau, M. M.; Valenzuela, R.; Ibarra-Soza, J.; Owens, H.; Fisher, A. J.; Beal, P. A. Controlling miRNA-like off-target effects of an siRNA with nucleobase modifications. *Organic & Biomolecular Chemistry* **2017**, *15* (47), 10029-10036, 10.1039/C7OB02654D. DOI: 10.1039/C7OB02654D.
(35) Huang, L.; Wang, J.; Lilley, D. M. J. Structure and ligand binding of the ADP-binding domain of the NAD+ riboswitch. *RNA* **2020**, *26* (7), 878-887. DOI: 10.1261/rna.074898.120.
(36) Matyjasik, M. M.; Hall, S. D.; Batey, R. T. High affinity binding of N2-modified guanine derivatives significantly disrupts the ligand binding pocket of the guanine riboswitch. *Molecules* **2020**, *25* (10), 2295. DOI: 10.3390/molecules25102295.
(37) Wang, B.; Zhang, T.; Yin, J.; Yu, Y.; Xu, W.; Ding, J.; Patel, D. J.; Yang, H. Structural basis for self-cleavage prevention by tag:anti-tag pairing complementarity in type VI Cas13 CRISPR systems. *Molecular Cell* **2021**, *81* (5), 1100-1115.e1105. DOI: https://doi.org/10.1016/j.molcel.2020.12.033.
(38) Beenstock, J.; Ona, S. M.; Porat, J.; Orlicky, S.; Wan, L. C. K.; Ceccarelli, D. F.; Maisonneuve, P.; Szilard, R. K.; Yin, Z.; Setiaputra, D.; et al. A substrate binding model for the KEOPS tRNA modifying complex. *Nature Communications* **2020**, *11* (1), 6233. DOI: 10.1038/s41467-020-19990-5.
(39) Balaratnam, S.; Torrey, Z. R.; Calabrese, D. R.; Banco, M. T.; Yazdani, K.; Liang, X.; Fullenkamp, C. R.; Seshadri, S.; Holewinski, R. J.; Andresson, T.; et al. Investigating the NRAS 5′ UTR as a target for small molecules. *Cell Chemical Biology* **2023**, *30* (6), 643-657.e648. DOI: https://doi.org/10.1016/j.chembiol.2023.05.004.
(40) Sun, Y.-T.; Varani, G. Structure of the dengue virus RNA promoter. *RNA* **2022**, *28* (9), 1210-1223. DOI: 10.1261/rna.079197.122.
(41) Chiu, L.-Y.; Emery, A.; Jain, N.; Sugarman, A.; Kendrick, N.; Luo, L.; Ford, W.; Swanstrom, R.; Tolbert, B. S. Encoded conformational dynamics of the HIV Splice Site A3 regulatory locus: Implications for differential binding of hnRNP splicing auxiliary factors. *Journal of Molecular Biology* **2022**, *434* (18), 167728. DOI: https://doi.org/10.1016/j.jmb.2022.167728.
(42) Chen, Y.; Tsai, B.; Li, N.; Gao, N. Structural remodeling of ribosome associated Hsp40-Hsp70 chaperones during co-translational folding. *Nature Communications* **2022**, *13* (1), 3410. DOI: 10.1038/s41467-022-31127-4.
(43) Zhang, X.; Li, S.; Pintilie, G.; Palo, M. Z.; Zhang, K. Snapshots of the first-step self-splicing of Tetrahymena ribozyme revealed by cryo-EM. *Nucleic Acids Research* **2023**, *51* (3), 1317-1325. DOI: 10.1093/nar/gkac1268 (acccessed 12/6/2024).
(44) Sampedro Vallina, N.; McRae, E. K. S.; Geary, C.; Andersen, E. S. An RNA paranemic crossover triangle as a 3D module for cotranscriptional nanoassembly. *Small* **2023**, *19* (13), 2204651. DOI: https://doi.org/10.1002/smll.202204651 (acccessed 2024/12/06).

(45) Vögele, J.; Duchardt-Ferner, E.; Bains, J. K.; Knezic, B.; Wacker, A.; Sich, C.; Weigand, Julia E.; Šponer, J.; Schwalbe, H.; Krepl, M.; et al. Structure of an internal loop motif with three consecutive U•U mismatches from stem–loop 1 in the 3′-UTR of the SARS-CoV-2 genomic RNA. *Nucleic Acids Research* **2024**, *52* (11), 6687-6706. DOI: 10.1093/nar/gkae349 (acccessed 12/6/2024).

(46) Vögele, J.; Hymon, D.; Martins, J.; Ferner, J.; Jonker, Hendrik R. A.; Hargrove, Amanda E.; Weigand, Julia E.; Wacker, A.; Schwalbe, H.; Wöhnert, J.; et al. High-resolution structure of stem-loop 4 from the 5′-UTR of SARS-CoV-2 solved by solution state NMR. *Nucleic Acids Research* **2023**, *51* (20), 11318-11331. DOI: 10.1093/nar/gkad762 (acccessed 12/6/2024).

(47) Menichelli, E.; Lam, B. J.; Wang, Y.; Wang, V. S.; Shaffer, J.; Tjhung, K. F.; Bursulaya, B.; Nguyen, T. N.; Vo, T.; Alper, P. B.; et al. Discovery of small molecules that target a tertiary-structured RNA. *Proceedings of the National Academy of Sciences* **2022**, *119* (48), e2213117119. DOI: 10.1073/pnas.2213117119 (acccessed 2024/12/06).

(48) Ma, S.; Kotar, A.; Grote, S.; Rouskin, S.; Keane, S. C. Structure of pre-miR-31 reveals an active role in Dicer processing. *bioRxiv* **2023**, 2023.2001.2003.519659. DOI: 10.1101/2023.01.03.519659.

(49) Gottipati, K.; McNeme, S. C.; Tipo, J.; White, M. A.; Choi, Kyung H. Structural basis for cloverleaf RNA-initiated viral genome replication. *Nucleic Acids Research* **2023**, *51* (16), 8850-8863. DOI: 10.1093/nar/gkad618 (acccessed 12/6/2024).

(50) Ding, J.; Deme, Justin C.; Stagno, J. R.; Yu, P.; Lea, Susan M.; Wang, Y.-X. Capturing heterogeneous conformers of cobalamin riboswitch by cryo-EM. *Nucleic Acids Research* **2023**, *51* (18), 9952-9960. DOI: 10.1093/nar/gkad651 (acccessed 12/6/2024).

(51) Escobar, C. A.; Petersen, R. J.; Tonelli, M.; Fan, L.; Henzler-Wildman, K. A.; Butcher, S. E. Solution structure of poly(UG) RNA. *Journal of Molecular Biology* **2023**, *435* (24), 168340. DOI: https://doi.org/10.1016/j.jmb.2023.168340.

(52) Nardini, M.; Gnesutta, N.; Donati, G.; Gatta, R.; Forni, C.; Fossati, A.; Vonrhein, C.; Moras, D.; Romier, C.; Bolognesi, M.; et al. Sequence-specific transcription factor NF-Y displays histone-like DNA binding and H2B-like ubiquitination. *Cell* **2013**, *152* (1), 132-143. DOI: https://doi.org/10.1016/j.cell.2012.11.047.

(53) Agalarov, S. C.; Sridhar, G.; Funke, P. M.; Stout, C. D.; Williamson, J. R. Structure of the S15,S6,S18-rRNA complex: Assembly of the 30s ribosome central domain. *Science* **2000**, *288* (5463), 107-112. DOI: 10.1126/science.288.5463.107 (acccessed 2024/12/06).

(54) Montemayor, E. J.; Didychuk, A. L.; Liao, H.; Hu, P.; Brow, D. A.; Butcher, S. E. Structure and conformational plasticity of the U6 small nuclear ribonucleoprotein core. *Acta Crystallographica Section D* **2017**, *73* (1), 1-8. DOI: doi:10.1107/S2059798316018222.
